# An Implantable Optogenetics‐Engineered Hydrogel for Amelioration of Rheumatoid Arthritis through Light‐Controlled Metabolic Reprogramming of Synovial Macrophages

**DOI:** 10.1002/advs.202523949

**Published:** 2026-05-12

**Authors:** Dahai Hu, Yaru Sun, Qi Lu, Yi Zhang, Jieruo Li, Huige Hou, Huajun Wang, Hui Tang, Yunsong Zhang, Xiaofei Zheng, Qingsong Mei

**Affiliations:** ^1^ Department of Sports Medicine, The First Affiliated Hospital, Guangdong Provincial Key Laboratory of Speed Capability, The Guangzhou Key Laboratory of Precision Orthopedics and Regenerative Medicine, School of Medicine Jinan University Guangzhou P. R. China; ^2^ Department of Medical Biochemistry and Molecular Biology, School of Medicine Jinan University Guangzhou Guangdong P. R. China; ^3^ The Clinical Medicine Research Institute The First Affiliated Hospital of Jinan University Guangzhou P. R. China; ^4^ The Clinical Medicine Research Institute The Fifth Affiliated Hospital of Jinan University Heyuan P. R. China; ^5^ The Department of Plastic and Reconstructive Surgery, The Affiliated Guangdong Second Provincial General Hospital Jinan University Guangzhou P. R. China

**Keywords:** cartilage damage, joint inflammation, macrophage polarization, metabolic reprogramming

## Abstract

Rheumatoid arthritis (RA) has always been a therapeutic challenge in clinical due to the lack of effective drug treatments. Inspired by the impact of daily diet on RA, herein we proposed an innovative energy metabolism modulation strategy to reprogram synovial macrophages for RA treatment. An implantable hydrogel, encapsulating with optogenetics‐engineered cells, was designed to enable in‐situ and on‐demand secretion of glucagon‐like peptide‐1 (GLP‐1) through blue light irradiation. GLP‐1 in synovium was found to activate GLP‐1R/HK2/VDAC1 pathway to upregulate the tricarboxylic acid cycle and oxidative phosphorylation in macrophages. This metabolic reprogramming elicited a phenotypic transition in macrophage polarization, shifting from M0/M1 state to M2 state. Significantly, the GLP‐1‐mediated approach reduced synovial inflammatory cytokine levels and facilitated tissue repair and bone erosion recovery. These findings reveal the therapeutic potential of GLP‐1R/HK2/VDAC1 pathway as a novel target for RA.

## Graphical abstract

An implantable hydrogel is designed to enable in situ and on‐demand secretion of GLP‐1 in synovium through blue light irradiation to activate GLP‐1R/HK2/VDAC1 pathway for upregulating tricarboxylic acid cycle and oxidative phosphorylation in macrophage, facilitating tissue repair and bone erosion recovery.

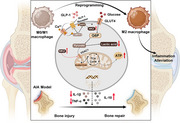

## 1 Introduction

Rheumatoid arthritis (RA) is a relatively common chronic inflammatory disease, which mainly affects the joints, meanwhile accompanying with extra‐articular manifestations, such as rheumatoid nodules, lung involvement, vasculitis and systemic comorbidities [1, 2]. The current treatment approach for RA primarily emphasizes non‐pharmacological interventions, such as physical therapy, rehabilitation exercises and dietary modifications, and is supplemented by conventional disease‐modifying anti‐rheumatic drugs, non‐steroidal anti‐inflammatory drugs, and glucocorticoids [1]. However, there are still many people, which is about 40% of RA patients, do not respond to current therapeutic strategies [3, 4, 5]. What's more unfortunate is that 5%–20% of patients are resistant to all existing drugs [6, 7]. Therefore, it is urgent to explore a safe and effective strategy for RA treatment.

RA is accompanied by synovial inflammation caused by immune activation, and macrophages play a significant role in the disease process of RA [8]. Macrophages can exhibit pro‐inflammatory M1 subtype or anti‐inflammatory M2 subtype under different environmental stimuli [9, 10]. M1 macrophages exacerbate cartilage destruction by secreting inflammatory cytokines, degenerative enzymes, and other factors, such as R‐spondin‐2 [11, 12, 13, 14, 15]. It was found that the M1/M2 ratio was abnormally elevated during the development of RA, and the balance between them coordinated the inflammatory and resolution phases after tissue damage [16, 17]. Reprogramming the naïve macrophages (M0) or M1 macrophages into M2 macrophages should provide a fertile soil for RA treatments. However, the complex in vivo microenvironment renders it difficult to achieve specific targeting of synovial macrophages, and conventional interventions are associated with the risks of off‐target effects and systemic immunosuppression [8, 18, 19]. Accordingly, the precise spatiotemporal regulation of macrophage polarization at the lesion site still poses a major challenge. Extensive evidence indicates that macrophages exhibit distinct metabolic characteristics among different cell subsets [20, 21, 22, 23]. M1 macrophages primarily rely on glycolytic metabolism, while M2 macrophages are distinguished by their energetic dependence on the tricarboxylic acid (TCA) cycle and oxidative phosphorylation [21, 24, 25]. Evidently, a daily diet with high contents of fat or sugar can aggravate RA. It is therefore anticipated that cellular reprogramming may be achieved by modulating the energy metabolic status of macrophages.

Glucagon‐like peptide‐1 (GLP‐1) is a multifunctional peptide hormone that plays a significant role in the regulation of energy metabolism. Recent studies have shown that GLP‐1 may play a protective role in the cardiovascular and nervous systems by reducing glycated hemoglobin, decreasing inflammation, and inhibiting cell apoptosis [26, 27]. Moreover, a clinical trial has shown that treatment with weekly injections of semaglutide (a GLP‐1 analogue) can significantly reduce knee pain in patients with obesity and knee osteoarthritis [28]. GLP‐1 recently was also reported to alleviate osteoarthritis inflammation through the gut‐joint axis [29, 30]. However, different from osteoarthritis, real‐time and dynamic regulation of local microenvironment is critical for RA treatments. Owing to its relatively short half‐life, the precise localized delivery of GLP‐1 at the lesion site of RA continues to pose a significant technical challenge. It still remains unknown whether GLP‐1 can alleviate RA.

**SCHEME 1:**
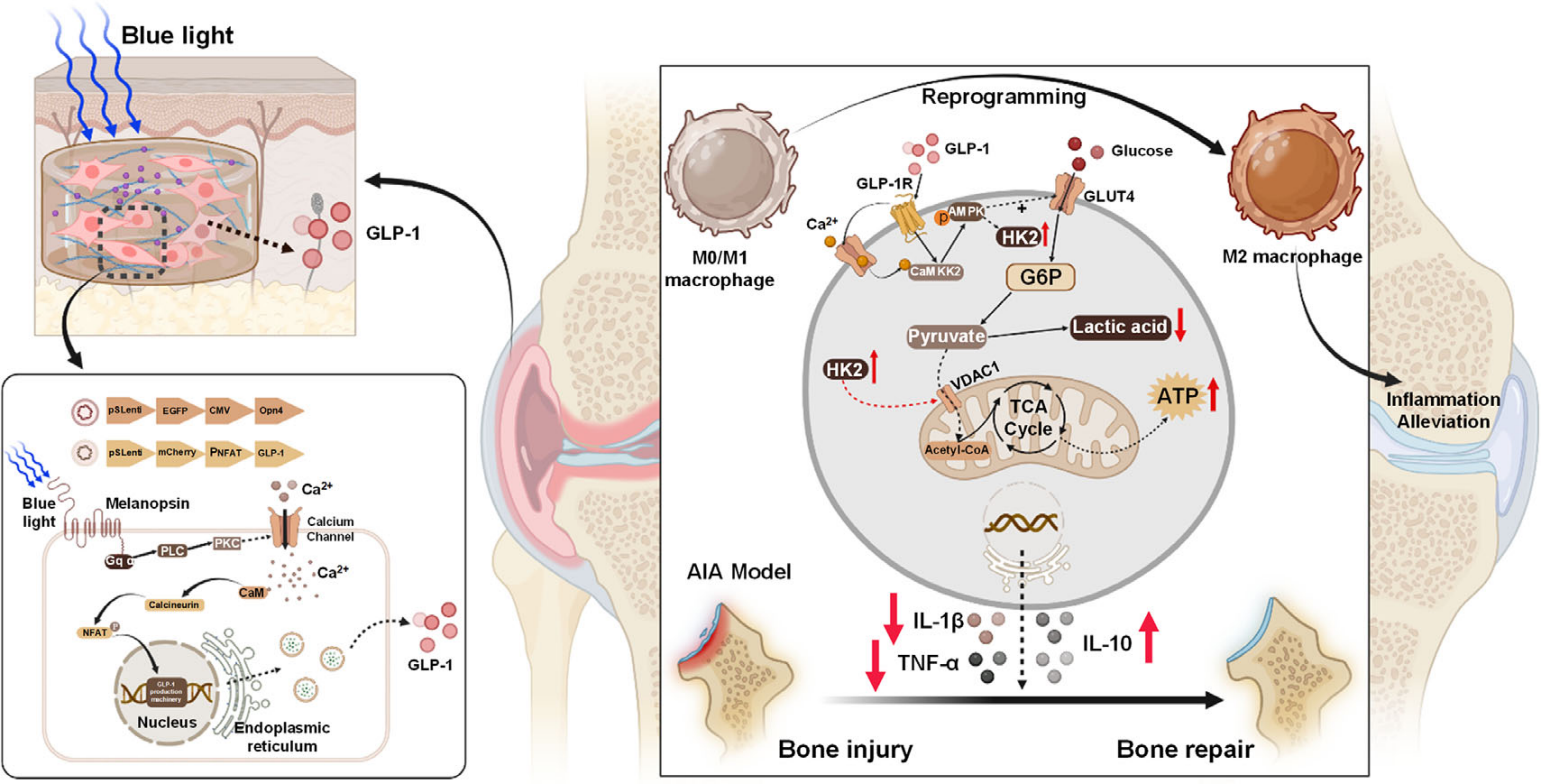
The cartoon illustrates that optogenetics‐engineered cells are encapsulated into hydrogel formulation for blue‐light controlled generation of GLP‐1 to alleviate RA symptoms. GLP‐1 regulated glucose metabolism by acting on the GLP‐1R/HK2/VDAC1 pathway to reprogram macrophages, and promote the transformation of M0/M1 to M2 phenotype, inhibiting the release of inflammatory factors and facilitating the repair of bone erosion. Figures were created with BioRender.com.

In this study, we developed an implantable hydrogel encapsulating opsins‐transfected HEK‐293 cells, which could in‐situ and tempo‐spatial precisely secrete GLP‐1 upon blue light irradiation. The hydrogel demonstrated significant therapeutic efficacy in a mouse model of RA, and exhibited favorable biosafety profiles. Mechanistic studies revealed that GLP‐1 activated the GLP‐1R on macrophages, regulated Ca^2+^ influx, and enhanced the expression of HK2 and VDAC1 proteins, thereby promoting tricarboxylic acid cycle and oxidative phosphorylation. This led to metabolic reprogramming and promoted M2 macrophages polarization, resulting in reduced inflammation and enhanced bone repair (Scheme 1). Our findings suggest that the GLP‐1R/HK2/VDAC1 pathway represents a promising therapeutic target for RA.

## 2 Results and Discussion

### 2.1 Design and Construction of the Optogenetics Therapy System

**FIGURE 1:**
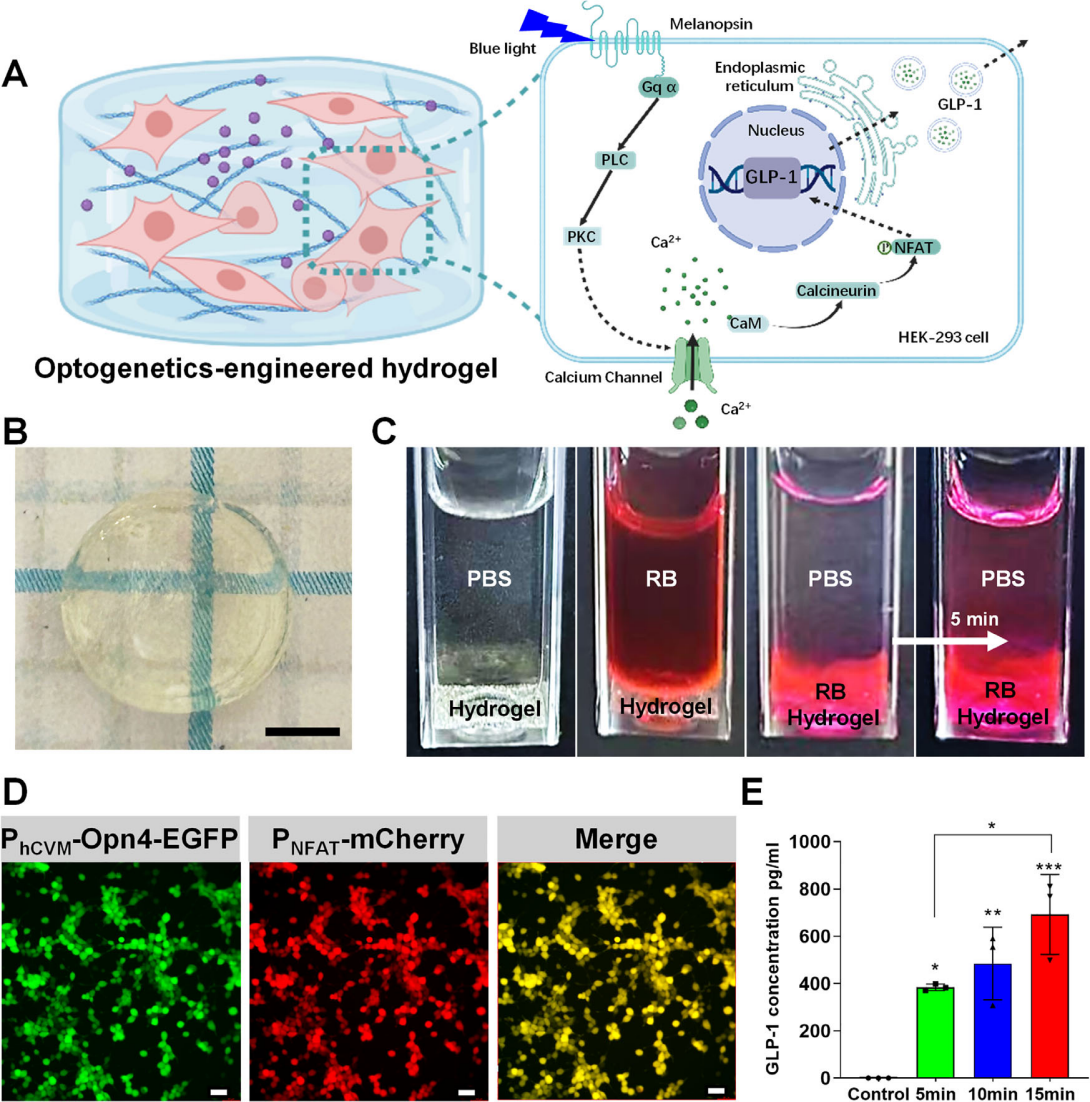
Construction of the optogenetics therapy system. (A) Schematic illustration of PEGDA hydrogel therapy system. (B) A photograph of the hydrogel. Scale bar is 1 mm. (C) Detecting the permeability of the hydrogel using Rhodamine B (RB). (D) Fluorescence images showed that the expression of EGFP protein (green) and mCherry protein (red) in the cells after transfection for 48 hours. Scale bars are 50 µm. (E) The expression level of GLP‐1 was detected after blue light irradiation. Data were expressed as mean ± SD, n = 3. Data were analyzed using one‐way ANOVA followed by Tukey's multiple comparisons test. *p < 0.05, **p < 0.01, ***p < 0.001.

We constructed an FDA approved biomaterial, polyethylene glycol diacrylate (PEGDA) hydrogel, as a scaffold to encapsulate the opsins‐transfected cells. As shown in Figure 1A, after blue light stimulation, the photosensitive protein melanopsin on the plasma membrane is activated, increasing Ca^2+^ influx, thereby activating the downstream transcription factor, nuclear factor of activated T cells (NFAT), to drive the production of GLP‐1, which has been well established in our previous works [31, 32, 33]. The obtained hydrogel possesses outstanding light‐penetrating capability, making it efficiently work for optogenetic activation (Figure 1B). To ensure its in vivo therapeutic applications, the implanted hydrogel must maintain structural integrity throughout the treatment period while facilitating the exchange of small molecules with the surrounding interstitial fluid. As shown in Figure S1, the implanted hydrogel maintained its structural integrity throughout the entire treatment period. The results in Figure 1C demonstrated that the fabricated hydrogel was capable of efficiently adsorbing small molecules such as rhodamine B (RB) and rapidly releasing them into the external fluid, as evidenced in Figure 1C.

Next, we examined whether the melanopsin expression vector P_hCMV_‐melanopsin‐EGFP and the P_NFAT_‐driven mCherry expression vector P_NFAT_‐mCherry were successfully co‐transfected into HEK‐293 cells. The fluorescence images showed that almost all HEK‐293 cells expressed green fluorescence produced by EGFP and red fluorescence produced by mCherry (Figure 1D). Therefore, HEK‐293 cells with a density of 250,000 cells/cm^2^ in PEGDA hydrogel were irradiated with blue light for 5, 10, and 15 min respectively to verify the controllable release of GLP‐1 in the supernatant (Figure 1E). The opsins‐transfected HEK‐293 cells were then encapsulated into PEGDA hydrogel to construct a therapeutic formulation for RA treatment (Figure S2). Cellular viability staining revealed that the opsins‐transfected HEK‐293 cells were still alive in the hydrogel even after 9 days incubation, indicating its excellent biocompatibility for the following applications (Figure S3).

### 2.2 GLP‐1 Reprogram M0/M1 to Anti‐Inflammatory M2 Macrophages

**FIGURE 2:**
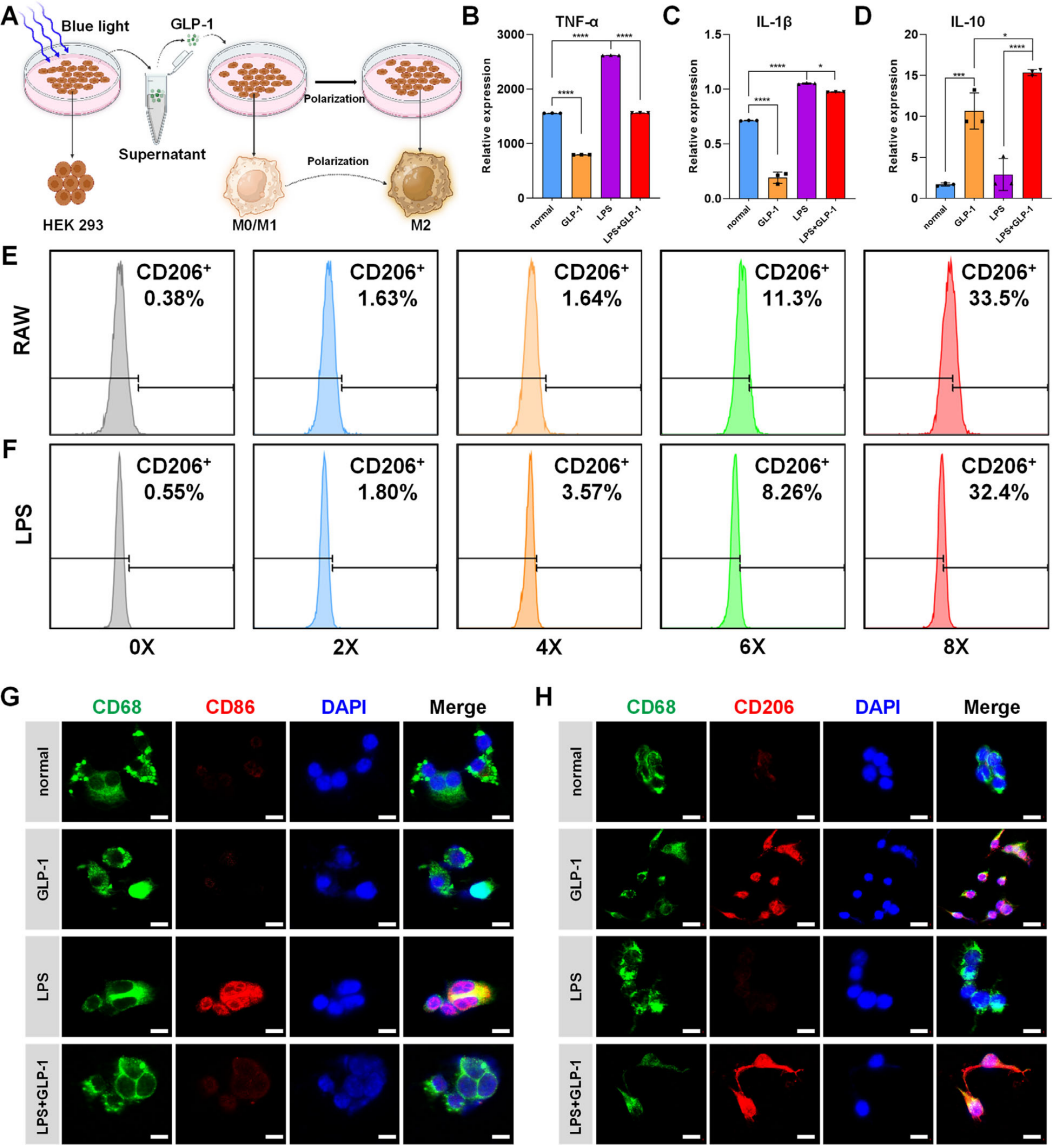
GLP‐1 reprograms M0/M1 to anti‐inflammatory M2 macrophages. (A) Schematic illustration of the secretion of GLP‐1 from hydrogel and the following interaction with macrophages. (B–D) The genes expressions of TNF‐α, IL‐1β (M1 phenotype) and IL‐10 (M2 phenotype) from RAW264.7 cells after different treatments. Data were expressed as mean ± SD, n = 3. Data were analyzed using one‐way ANOVA followed by Tukey's multiple comparisons test. *p < 0.05, ***p < 0.001, ****p < 0.0001. (E, F) The proportion of M2 phenotype macrophages (CD206^+^) in RAW264.7 cells and RAW264.7 cells pretreated with LPS was detected by flow cytometry. (G, H) The confocal microscopy images showed that after treatment with GLP‐1, the expression of CD206 was enhanced and the expression of CD86 was weakened in RAW264.7 cells. Scale bars are 20 µm.

Since GLP‐1 can regulate the glycometabolism pathway and inhibit inflammation [34, 35], it is expected to explore its role in macrophage polarization. Herein, the mouse macrophage cell line RAW264.7 was used to demonstrate the re‐programmability of GLP‐1 (Figure 2A). It was found that GLP‐1 significantly reduced the expression levels of the M1 markers TNF‐α and IL‐1β, and greatly increased the expression level of the M2 marker IL‐10, whether the cells were pretreated with lipopolysaccharide (LPS) or not (Figure 2B–D). It is well known that the metabolism of M1 macrophages mainly relies on glycolytic, accompanying with the release of lactate, while M2 macrophages mainly relies on oxidative phosphorylation [36]. The treatment with GLP‐1 was expected to enhance the TCA cycle and oxidative phosphorylation of macrophages, thus improving the proportions of M2 macrophages. The results of flow cytometry revealed that the proportions of M2 macrophages which were labelled with CD206 increased by 60–90 folds with the increase of GLP‐1 stimulation concentration (Figure 2E, F).

Furthermore, immunofluorescence staining for CD68 (the pan macrophage marker), CD86 (M1 marker), and CD206 (M2 marker) was exploited to verify this transformation. As expected, a significant increase in CD86 was observed in LPS pretreated RAW264.7 cells (Figure 2G). After GLP‐1 stimulation, the fluorescence intensity of CD86 decreased, while the fluorescence intensity of CD206 increased in both RAW264.7 cells and LPS‐pretreated RAW264.7 cells (Figure 2H; Figures S4–S7). These data solidly indicate that GLP‐1 can effectively promote the reprogramming of M0/M1 macrophages to M2 macrophages.

### 2.3 GLP‐1 Inhibits Inflammation and Promotes Bone Repair in AIA Mice

**FIGURE 3:**
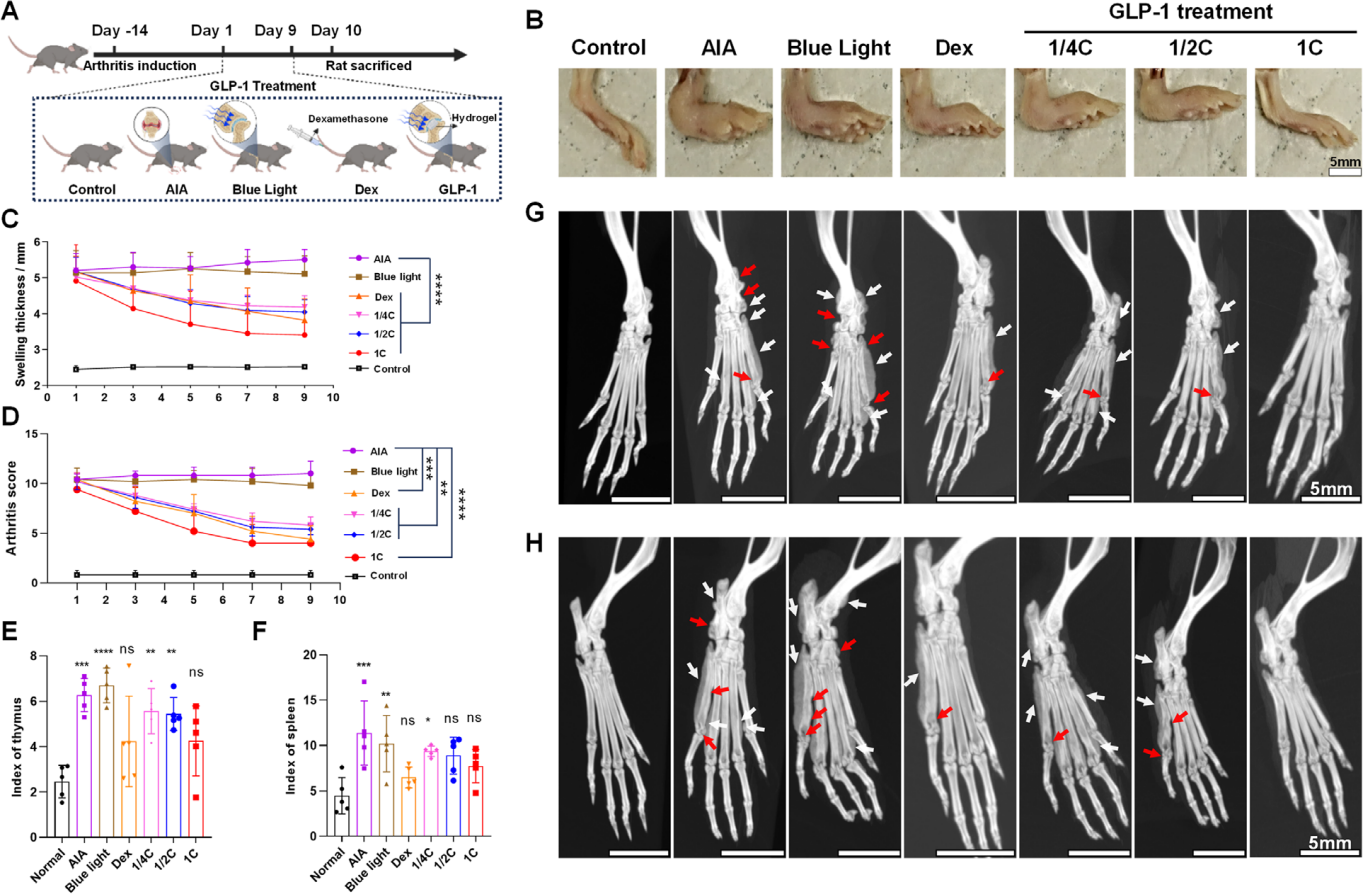
GLP‐1 significantly alleviates the symptoms of RA. (A) A schematic illustration of GLP‐1 treatment of AIA mice. (B) Representative images of the hind limbs in different groups at the end of treatments. Scale bars are 5 mm. (C, D) The paw thickness and arthritis score of AIA mice were recorded every two days during the treatment period. Data represent mean ± SD (n = 5). Data were calculated by two‐way ANOVA. **p < 0.01, ***p < 0.001, ****p < 0.0001. (E, F) The index of spleen and thymus of AIA mice were recorded at the end of treatment. Data represent mean ± SD (n = 5). Data were calculated by the unpaired two‐tailed Student's t‐test. *p < 0.05 vs. normal, **p < 0.01 vs. normal, ***p < 0.001 vs. normal, ****p < 0.0001 vs. normal. Representative micro‐CT images of the anterolateral (G) and the posterolateral (H) ankle joints at the end of treatment for different groups. Scale bars are 5 mm. Red arrows indicate bone erosion, and white arrows indicate inflammatory hyperplasia.

An antigen‐induced arthritis (AIA) mouse model was constructed to further investigate therapeutic effects of GLP‐1 for RA diseases (Figure 3A). All the animal experiments in this study were conducted in accordance with the national regulations on laboratory animals in China, and were approved by the Institutional Animal Care and Use Committee of Jinan University (IACUC‐20241126‐20). A glucocorticoid, dexamethasone (Dex), which is a commonly used clinical drug with potent anti‐inflammatory and analgesic effects, was used as a pharmacological control to compare the treatment effects with GLP‐1 treatments. For the Dex group, treatment was administered via tail vein injection of 0.05 mL/g Dex solution (concentration: 1 mg/mL), with the frequency of given one time for every three days. In the GLP‐1 group, the hydrogel formulation was placed subcutaneously in the mouse joint and irradiated with blue light, with irradiation twice daily for 15 min of each time. The entire treatment period lasted for nine days. Additionally, the GLP‐1 treatment groups were divided into three subgroups with different cell amounts in the hydrogel, named with 1C (930,000 cells), 1/2C (460,000 cells), and 1/4C (230,000 cells). Finally, there were no significant differences in the body weights of mice in each group before and after 9 days treatment (Figure S9).

As shown in Figure 3B and Figure S10, both GLP‐1 and Dex exhibited potent therapeutic effects, obviously reducing paw swelling and markedly alleviating the progression of the disease in AIA mice. Furthermore, the paw swelling thickness was measured, and arthritis scores of AIA mice was calculated to quantitatively reveal treatment effects (Figure 3C, D). It was found that the paw swelling thickness of blue‐light illumination group showed negligible difference from AIA group. GLP‐1 group with maximum cell amounts demonstrated excellent treatment effects on both paw swelling and arthritis scores. From the perspective of immunology, compared with normal mice, the spleen and thymus index of the AIA group and blue‐light group were significantly increased, while those in the GLP‐1 and Dex groups were significantly decreased (Figure 3E, F). It indicates that after treatment with GLP‐1 or Dex, the inflammation related immune response can be effectively reduced. Furthermore, the bone erosion was further verified through Micro‐CT analysis. At the end of the treatment, the AIA group and blue‐light group exhibited severe bone erosion and inflammatory hyperplasia, while the bone erosion and inflammatory hyperplasia in the GLP‐1 group and Dex group were significantly alleviated (Figure 3G, H). While the Dex group exhibits nearly comparable therapeutic efficacy to the GLP‐1 group, the prolonged administration of Dex is associated with well‐documented adverse clinical effects, including osteoporosis, fractures, Cushing's syndrome, and immunosuppression, among others [37, 38, 39]. In contrast, GLP‐1 treatment represents a safer therapeutic modality.

**FIGURE 4:**
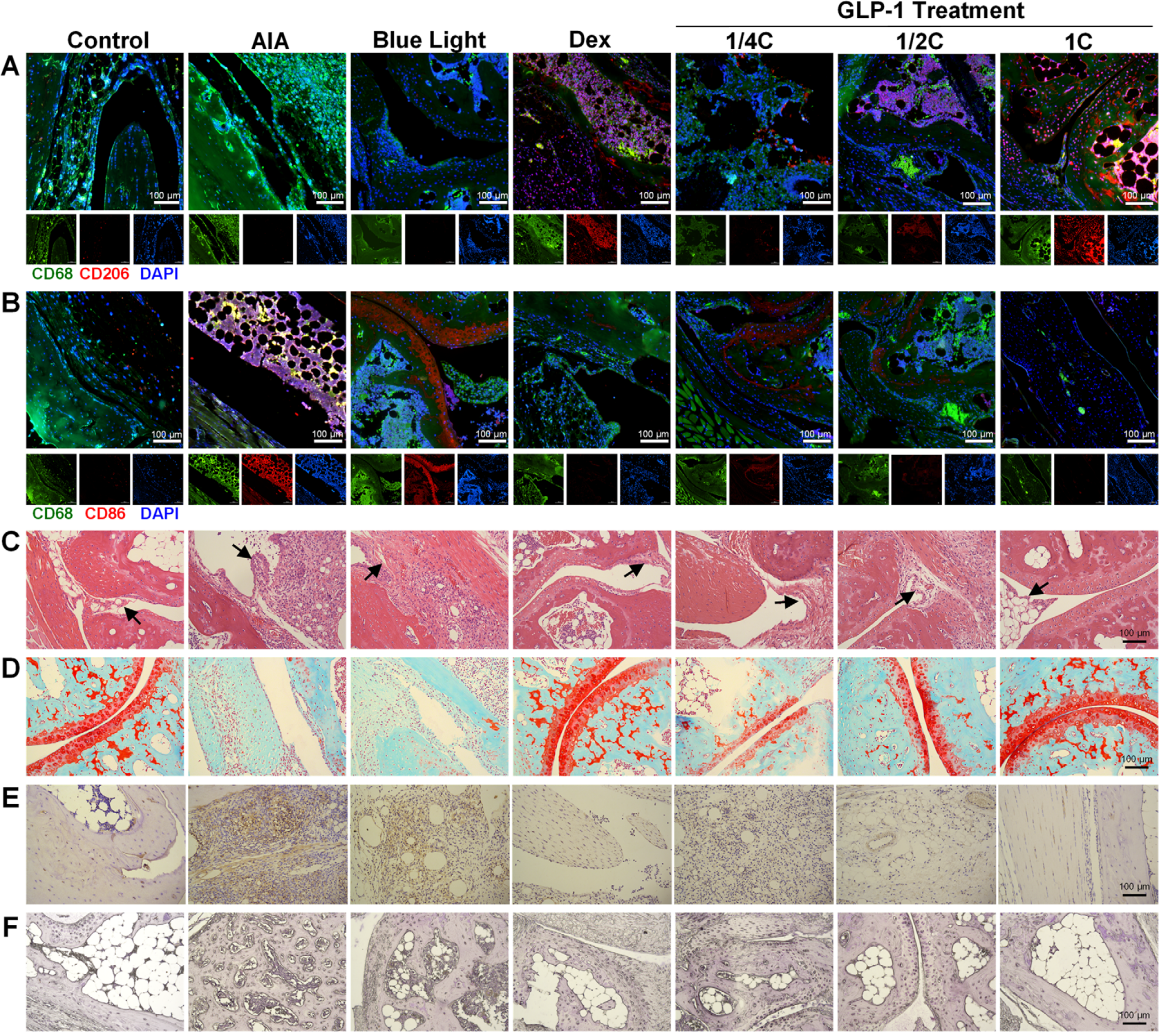
GLP‐1 inhibits inflammation and promotes bone repair in AIA mice. (A, B) The immune fluorescence images showed the expression levels of CD206 (red) and CD86 (red) in the joint sections of mice from different groups. Macrophages were labeled with CD68 antibody (green). Cell nucleus was stained with DAPI (blue). Scale bars are 100 µm. (C) Histopathology evaluation of ankle joints was identified using H&E. Scale bars are 100 µm. (D) Safranin O‐Fast green staining was used to detect the degree of cartilage damage in mice. Scale bars are 100 µm. (E) Immunohistochemical analyses of IL‐1β expression levels in mice joints in different groups. Scale bars are 100 µm. (F) Detection of TRAP‐stained osteoclast expression levels in arthritic joints in different groups. Scale bars are 100 µm.

The ankle joints were collected and sliced into thin sections for immunofluorescence staining of CD68, CD86, CD206, and DAPI after the treatments to demonstrate whether GLP‐1 can effectively inhibit the progression of joint inflammation and cartilage damage in AIA mice and actively promote the repair of bone erosion. Compared with the AIA group and blue‐light group, the fluorescence intensity of CD206 was significantly increased in the GLP‐1 group and Dex group (Figure 4A). In contrast, the fluorescence intensity of CD86 was significantly increased in the AIA group and blue‐light group, and it was decreased in the GLP‐1 group and Dex group (Figure 4B). These results verified that GLP‐1 could also increase the proportion of anti‐inflammatory M2 macrophages in the inflamed tissues of AIA mice and decrease the proportion of pro‐inflammatory M1 macrophages.

H&E‐stained sections in Figure 4C showed massive infiltration of inflammatory cells and severe synovial hyperplasia in the AIA and blue‐light groups, while the GLP‐1group and Dex treatments could significantly alleviate synovial inflammation. Safranin‐O Fast‐Green staining showed that the cartilage and bone tissues in the joint sections of the AIA group and blue‐light group were mostly lost. In contrast, in the joints of GLP‐1 group and Dex group, red and intact cartilage was visible, indicating that GLP‐1 could effectively alleviate cartilage lesions in the joints (Figure 4D). Furthermore, immunohistochemical analysis showed that GLP‐1 effectively downregulated the secretion of the inflammatory cytokine IL‐1β (Figure 4E). Finally, we evaluated the effects of GLP‐1 on the expression levels of related markers using tartrate‐resistant acid phosphatase (TRAP)‐stained osteoclasts. The results showed that the AIA group and blue‐light group had increased numbers of osteoclasts and bone erosion in the ankle joints of mice, while the GLP‐1 group and Dex group had reduced numbers of osteoclasts and promoted bone damage repair (Figure 4F). The switch of macrophages from M1 to M2 phenotype appeared to prevent osteoclast‐related bone resorption and make the natural repair of the damaged bone possible.

### 2.4 GLP‐1 Reprograms Macrophages by Regulating Energy Metabolism

**FIGURE 5:**
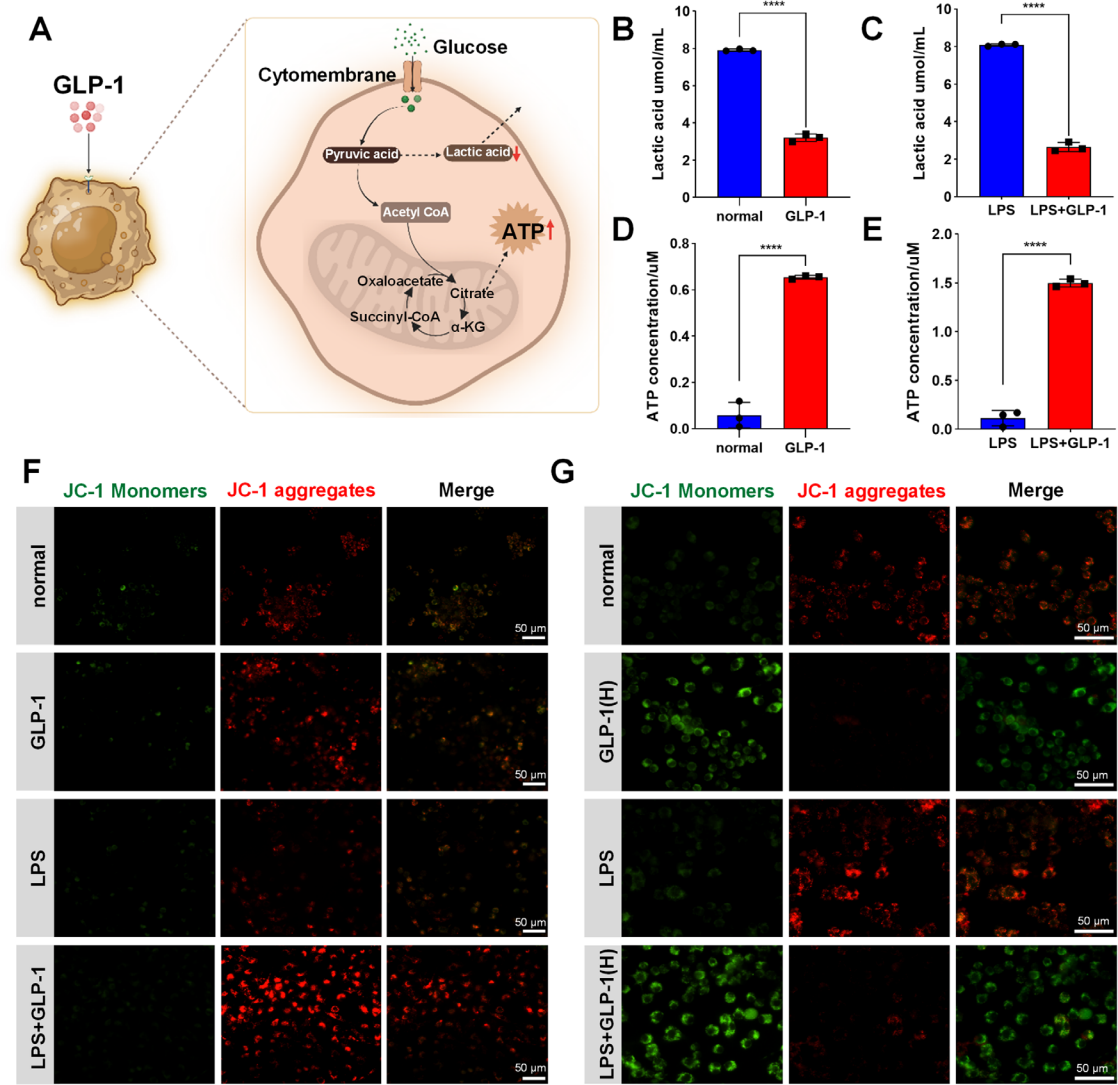
GLP‐1 reprograms macrophages by regulating energy metabolism. (A) A schematic illustration of GLP‐1 regulating glucose metabolism in macrophages. (B–E) The expressions of lactic acid and ATP were detected in RAW264.7 cells after different treatments. Data were expressed as mean ± SD, n = 3. Data were calculated by the unpaired two‐tailed Student's t‐test. ****p < 0.0001. (F) The mitochondrial membrane potential was detected in RAW264.7 cells after different treatments. JC‐1 monomers (green) represented the decrease in membrane potential, and JC‐1 aggregates (red) represented the increase in membrane potential. (G) After high concentration GLP‐1 treatment, the mitochondrial membrane potential was detected in RAW264.7 cells.

Given the ability of GLP‐1 to regulate glucose metabolism, it is speculated that macrophages reprogram is ascribed to the regulation of energy metabolism of macrophages (as shown in Figure 5A). The energy metabolic status of macrophages was analyzed by measuring the levels of lactate and ATP. It was found that RAW264.7 cells exhibited reduced lactate release and increased ATP content after treatment with GLP‐1 (Figure 5B, C). Similarly, RAW264.7 cells pretreated with LPS also showed decreased lactate release and increased ATP content (Figure 5D, E). Therefore, GLP‐1 can effectively inhibit glycolysis while enhancing the TCA cycle and oxidative phosphorylation, thereby regulating energy metabolism to reprogram macrophages.

It is well known that the mitochondrial membrane potential is closely related to ATP release due to protons’ entrance into the mitochondria intermembrane space during the process of TCA cycle and oxidative phosphorylation. Thus, we further detected mitochondrial membrane potential by use of JC‐1 dyes to examine the cellular energy metabolic status. The bright red fluorescence in RAW264.7 cells indicated its membrane potential rose after treating with GLP‐1 (Figure 5F), proving the increasing ATP synthesis. However, we found that high concentration of GLP‐1 for single‐time treatment greatly increased green fluorescence intensities in RAW264.7 cells, indicating the decrease of mitochondrial activity and increase of cell apoptosis (Figure 5G; Figure S11). The results showed that high concentration of GLP‐1 stimulation would exert negative influence on RA therapy. Therefore, it is better to treat with small amounts of GLP‐1 for multiple times during RA therapies.

### 2.5 GLP‐1 Reprograms Macrophages Through the HK2/VDAC1 Pathway

**FIGURE 6:**
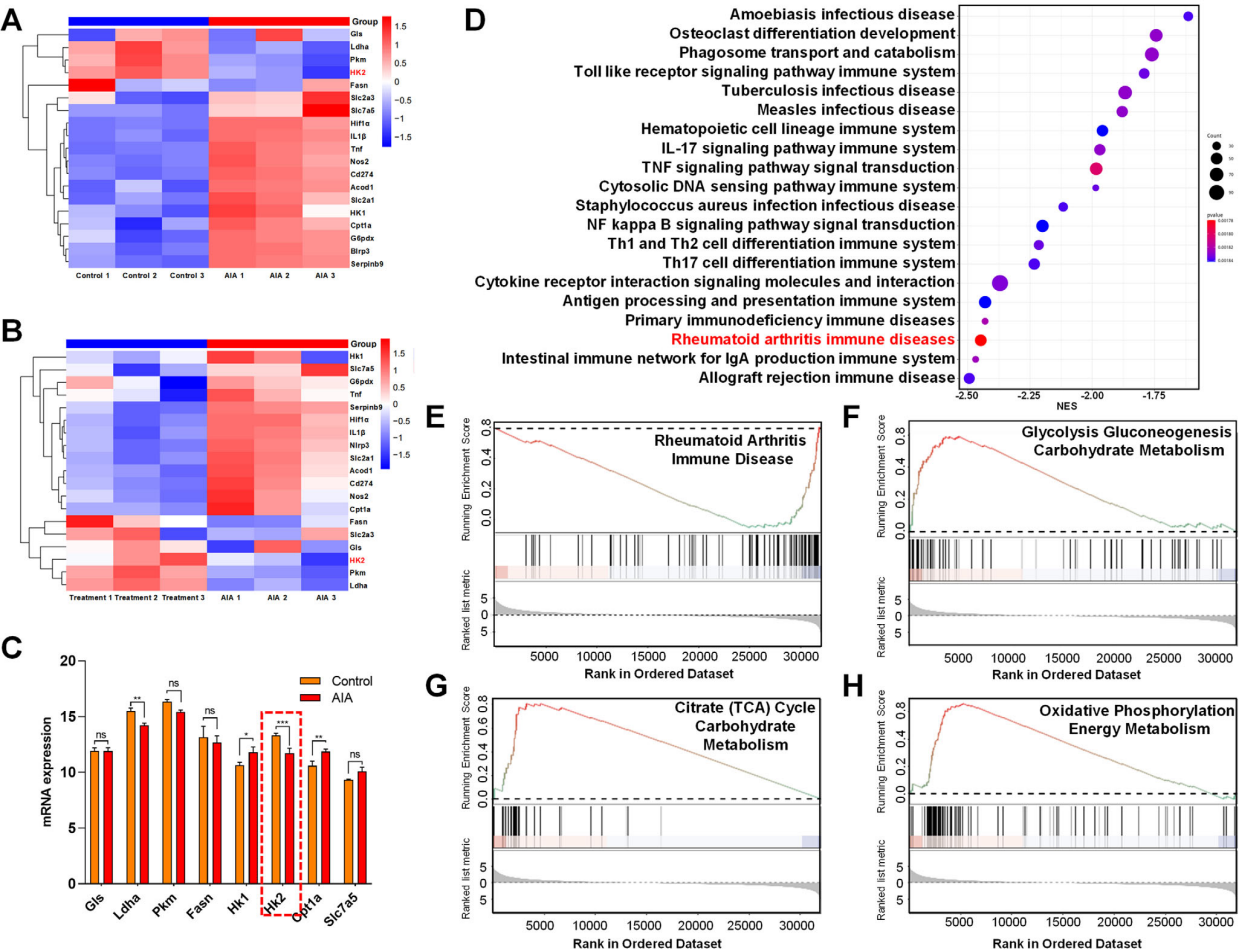
Transcriptomic analysis of ankle joint samples after GLP‐1 treatments. (A–C) Heatmap shows the differences in mRNA expression levels between AIA group and normal group, as well as the levels between AIA group and GLP‐1 group. Data in (C) were expressed as mean ± SD, n = 3. Data were calculated by the unpaired two‐tailed Student's t‐test. ^ns^ *P* > 0.05, *p < 0.05, **p < 0.01, ***p < 0.001. (D–H) KEGG and GSEA enrichment analyses were performed to explore the biological process.

Although GLP‐1 is known to reprogram macrophages through energy metabolism in above studies, the detailed mechanism is still illusive. To further explore the underpinning of GLP‐1 mediated re‐programmability, we collected ankle joint samples from normal group, AIA group, blue‐light group, and GLP‐1 group of mice, and performed transcriptomic analysis. The heatmap results based on the transcriptomic data showed that compared with AIA group, the Hexokinase 2 (HK2) protein was significantly upregulated in the GLP‐1 group and normal group (Figure 6A–C; Figure S12). The HK2 protein, as a key initial enzyme in glycolysis, plays a significant role in the regulation of cellular glucose metabolism [40]. Through KEGG and GSEA enrichment analyses, we explored the signaling pathways related to the enrichment of differentially expressed genes (DEGs). These analyses revealed that the most significant DEGs enrichment occurred in pathways related to RA immune response, glycolysis gluconeogenesis carbohydrate metabolism, TCA cycle carbohydrate metabolism, and oxidative phosphorylation energy metabolism (Figure 6D–H). HK2 is an essential metabolic enzyme and a key enzyme for phosphorylating glucose to G‐6‐P. By binding to voltage‐dependent anion channels (VDACs), HK2 promotes TCA cycle and oxidative phosphorylation and increases the frequency of G‐6‐P entering the glycolytic pathway, thereby reducing the formation of lactic acid and the pentose phosphate pathway [40, 41]. Therefore, GLP‐1 appears to reprogram macrophages by regulating glucose metabolism through HK2.

**FIGURE 7:**
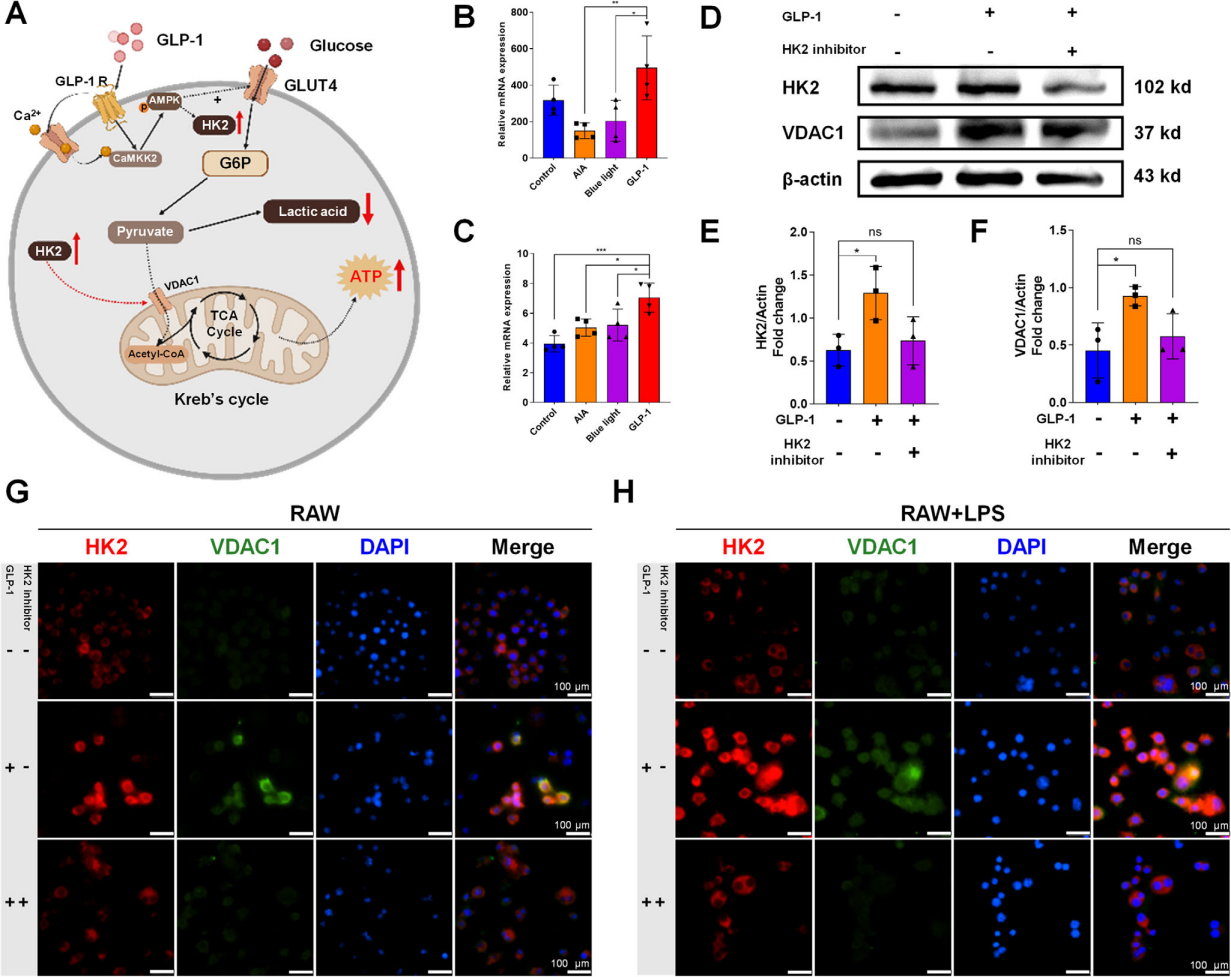
GLP‐1 reprograms macrophages through the HK2/VDAC1 pathway. (A) A schematic illustration of the mechanism of GLP‐1 reprogramming macrophages. (B, C) RNA was extracted, and qPCR was performed to measure endogenous mouse HK2 and VDAC1 mRNA levels. Data were expressed as mean ± SD, n = 4. Data were analyzed using one‐way ANOVA followed by Tukey's multiple comparisons test. *p < 0.05, **p < 0.01, ***p < 0.001. (D–F) Western blot analysis was performed to measure the levels of HK2 and VDAC1 proteins. The RAW264.7 cells were treated with an HK2 inhibitor. Data were expressed as mean ± SD, n = 3. Data were calculated by one‐way ANOVA followed by Tukey's multiple comparisons test. *p < 0.05. (G‐H) The immune fluorescence images show the expression levels of HK2 (red) and VDAC1 (green) in RAW264.7 cells after different treatments. Scale bars are 100 µm.

Based on above observation, we further investigated how HK2 regulates the energy metabolism process (Figure 7A). As we known, voltage‐dependent anion channel 1 (VDAC1) is an abundant channel protein in the mitochondrial outer membrane and is closely related to HK2 in cellular energy metabolism [42]. Therefore, we used RT‐qPCR to examine their expression levels, and found that the expression of HK2 and VDAC1 was significantly higher in the GLP‐1 group compared with AIA group and blue‐light group (Figure 7B, C). Subsequently, we employed an HK2 inhibitor to suppress the expression of HK2. The western blotting results indicated that following GLP‐1 treatment, the protein expression of HK2 and VDAC1 was significantly increased. However, upon the addition of the HK2 inhibitor, the expression of HK2 and VDAC1 decreased (Figure 7D–F).

Immunofluorescence experiments were also utilized to investigate the effects of GLP‐1 on the expression of HK2 and VDAC1. After GLP‐1 treatment, an obvious Ca^2+^ influx was found in RAW cells no matter whether it was treated with LPS (Figure S13), and then the fluorescence intensity of HK2 and VDAC1 was significantly enhanced. Conversely, after treatment with the HK2 inhibitor, the fluorescence intensity of HK2 and VDAC1 decreased regardless of macrophages were treated with LPS or not (Figure 7G, H). These results indicate that after GLP‐1 acting with its receptor (GLP‐1R), it induced the phosphorylation of AMPK through Ca^2+^‐mediated activation of CaMKK2 [43], thereby upregulating the expression of HK2 and VDAC1. TCA cycle and oxidative phosphorylation were followed to be promoted, and then macrophages were reprogrammed to alleviate inflammation.

## 3 Conclusions

In this study, we sought to address the limitation of GLP‐1's short half‐life by engineering an optogenetics‐based hydrogel capable of controlling the release of GLP‐1 in the bone joint cavity. This approach demonstrated promising anti‐rheumatoid arthritis therapeutic effects. The in‐situ release of GLP‐1 was found to polarize M0/M1 macrophages into the M2 phenotype, thereby inhibiting the expression of inflammatory factors and promoting tissue and bone erosion repair. In RA patients, secondary osteoporosis is prevalent due to the increased activity of osteoclasts, which disrupts the balance between osteoclasts and osteoblasts, exacerbating bone resorption. The observed shift from M1 to M2 macrophage phenotypes appears to prevent osteoclast‐mediated bone resorption, facilitating the natural repair of damaged bone. Furthermore, M2 macrophages enhance tissue remodeling in RA by secreting anti‐inflammatory cytokines. Additionally, our findings revealed that GLP‐1 regulates glucose metabolism via the HK2/VDAC1 pathway, which reprograms macrophage activity. These results suggest that the HK2/VDAC1 pathway may serve as a novel therapeutic target for the development of drugs targeting RA‐related pathologies.

## Author Contributions

**Q.M**., **X.Z**., and **H.T**. conceived the project and designed the research. **D.H**. performed the experiments and wrote the first draft. **Y.S**., **Q.L**., and **Y.Z**. performed the plasmids transfection. **J.L**., **H.H**., and **H.W**. analyzed the data and edited the manuscript. **Q.M**., **X.Z**., and **Y.Z**. provided essential reagents and funding. All authors approved the manuscript.

## Conflicts of Interest

The authors declare no conflicts of interest.

## Supporting information




**Supporting File**: advs75672‐sup‐0001‐SuppMat.docx.

## Data Availability

The data that support the findings of this study are available from the corresponding author upon reasonable request.
