# Supplementary material for: An Implantable Optogenetics‐Engineered Hydrogel for Amelioration of Rheumatoid Arthritis through Light‐Controlled Metabolic Reprogramming of Synovial Macrophages
Source: Adv Sci (Weinh). 2026 May 12:e23949. Online ahead of print. doi: 10.1002/advs.202523949 (PMC13336046; doi:10.1002/advs.202523949)
Supplement: Supplementary file 1 — Supporting File: advs75672‐sup‐0001‐SuppMat.docx. [file ADVS-9999-e23949-s001.docx]

Supporting Information

An Implantable Optogenetics-Engineered Hydrogel for Amelioration of Rheumatoid Arthritis through Light-Controlled Metabolic Reprogramming of Synovial Macrophages

Dahai Hu, Yaru Sun, Qi Lu, Yi Zhang, Jieruo Li, Huige Hou, Huajun Wang,* Hui Tang,* Yunsong Zhang,* Xiaofei Zheng,* Qingsong Mei*

**Materials and Methods**

**Animal and Cell**

Healthy male BALB/c mice (21-26 g, 9-10 weeks old) were purchased from the Zhuhai Bestest Bio-Tech Co., Ltd. (Zhuhai, China). The animal experiments in this study were conducted in accordance with the national regulations on laboratory animals in China and were approved by the Institutional Animal Care and Use Committee of Jinan University (IACUC-20241126-20). The HEK-293 and Raw 264.7 cell line were cultured in Dulbecco’s modified Eagle’s medium (DMEM), with 100 mg/L puromycin, 10% fetal bovine serum, and 100 U/mL penicillin. The cells were incubated in a 37 °C incubator with 5% CO2.

**Obtain Stably Co-transfected HEK-293 cells**

**1.1 Plasmid purification, transformation, and expansion**

Design the plasmids pSLenti-EF1-EGFP-P2A-Puro-CMV-Opn4-3xFLAG-WPRE and pSLenti-EF1-mCherry-P2A-Puro-PNFAT-GLP-1-3xFLAG-WPRE. The specific preparation process is as follows:

Step 1: The pSLenti-EF1-EGFP-P2A-Puro-CMV-Opn4-3xFLAG-WPRE plasmid was constructed by inserting the Opn4 gene (GenBank ID: NM_013887.2) into the MCS site of the empty vector plasmid GL107 pSLenti-EF1-EGFP-P2A-Puro-CMV-MCS-3xFLAG-WPRE. The synthesis steps were completed by Obio Technologies Corp., Ltd.

Step 2: For the pSLenti-EF1-mCherry-P2A-Puro-PNFAT-GLP-1-3xFLAG-WPRE plasmid, it was constructed based on the pSLenti-EF1-EGFP-P2A-Puro-CMV-MCS-3xFLAG-WPRE plasmid by knocking out the CMV promoter and replacing it with the NFAT-GLP-1-Linker-IgG-FC fusion protein sequence. Additionally, the sequence of the EGFP fluorescent protein was replaced with the sequence of the mCherry fluorescent protein. The synthesis steps were all carried out by Gene Optimal Biotechnology.

**1.2 Lentivirus Transfection**

The HEK-293 cells were seeded in six-well plates at approximately 5×10⁵ cells per well and cultured for 16-24 hours. Before transfection, the cells were subjected to starvation treatment for 6 hours. Subsequently, lip3000 was mixed with the plasmids prepared in ***Section 1*** using Opti-MEM. The target plasmid and packaging plasmids (psPAX2, pMD2.G) were added to the HEK-293 cells at a ratio of 4:3:1, with a total plasmid concentration of 4 µg per well. The transfection was performed using the Lip3000 transfection reagent according to the manufacturer's instructions, and subsequent experiments were carried out after the transfection was completed.

**1.3 Verification of Plasmid Transfection by Fluorescence Imaging**

The HEK-293 cells transfected with the dual plasmids in ***Section 1.2*** were seeded into six-well plates. After 48 hours of transfection, the expression of EGFP and mCherry proteins in the cells was observed through the green and red fluorescence channels of the inverted immunofluorescence microscope (Eclipse Ti2, Nikon, Japan), respectively.

**Preparation of the hydrogel**

200 μL of 10% w/v PEGDA PBS solution was mixed with 4 mg of Irgacure photoinitiator. The mixture was vortexed until a clear solution formed, then transferred to a mold (dimensions: pore diameter 0.3 cm × pore depth 0.7 cm) and cured under UV light for 15 minutes.

**Permeability of the hydrogel**

Cured the hydrogel at the bottom of the column. Added diluted rhodamine dye solution above the hydrogel and observed the color change of the gel. Then removed the supernatant liquid and washed five times with PBS. Subsequently, added PBS solution above the hydrogel and observed the color change of the supernatant liquid after 5 minutes.

**Measurement of GLP-1 Expression**

The expression of GLP-1 was measured using a Human Glucagon-Like Peptide 1 (GLP-1, 7-37) ELISA Kit (ELK5218, ELK Biotechnology) according to the manufacturer's instructions. The transfected HEK-293 cells were seeded into six-well plates at approximately 6×10⁵ cells per well and cultured for 16-24 hours. Before blue light irradiation, the culture medium of the HEK-293 cells was replaced with fresh medium. After 5 minutes of uniform blue light irradiation, 200 µL of the supernatant was collected for measurement (GLP-1 release at 5 minutes). After another 5 minutes of uniform blue light irradiation, 200 µL of the supernatant was collected for measurement (GLP-1 release at 10 minutes). After an additional 5 minutes of uniform blue light irradiation, 200 µL of the supernatant was collected for measurement (GLP-1 release at 15 minutes).

In the ELISA procedure, 50 µL of standard working solution or 50 µL of sample was added to each well of the microplate at room temperature, followed immediately by the addition of 50 µL of biotinylated conjugate working solution. The plate was incubated at 37℃ for 60 minutes, and then the liquid was discarded. Each well was washed three times with 200 µL of wash buffer. After patting dry, 100 µL of HRP enzyme working solution was added to each well and incubated at 37 ℃ for 60 minutes. The liquid in the plate was discarded again, and each well was washed with 200 µL of wash buffer, repeated five times. After patting dry, 90 µL of TMB was added to each well and incubated at 37 ℃ for 20 minutes. 50 µL of stop solution was added to each well, and the absorbance was measured at 450 nm. The results were calculated based on the standard curve.

**Measurement of TNF-α, IL-1β, and IL-10 Expression**

The expression levels of TNF-α, IL-1β, and IL-10 cytokines in the supernatant were measured using the TNF-α ELISA Kit (EM008-16, Excell), IL-1β ELISA Kit (EM001-96, Excell), and IL-10 ELISA Kit (EM005-96, Excell), respectively.

The RAW264.7 cells were seeded into six-well plates at approximately 1×10⁶ cells per well and cultured for 24 hours at 37 ℃ with or without LPS (200 ng/mL) stimulation. The transfected HEK-293 cells were also seeded into six-well plates at approximately 1×10⁶ cells per well and cultured for 16-24 hours. Before blue light irradiation of the transfected HEK-293 cells, the culture medium was replaced with fresh medium. After 15 minutes of blue light irradiation, the supernatant was immediately collected and added to the RAW264.7 cells, followed by incubation for 8 hours. The supernatant of the RAW264.7 cells was then collected for ELISA detection.

For the ELISA procedure, 100 µL of standard solutions at different concentrations or samples were added to the corresponding wells and incubated at 37 ℃ for 90 minutes. The liquid in the wells was then discarded, and the plate was washed five times with wash buffer. Subsequently, 100 µL of biotinylated antibody working solution was added to each well and incubated at 37 ℃ for 60 minutes. The liquid was discarded again, and the plate was washed five times. Next, 100 µL of enzyme conjugate working solution was added to each well and incubated at 37 ℃ for 30 minutes. The liquid was discarded once more, and the plate was washed five times. Then, 100 µL of substrate solution was added to each well and incubated at 37 ℃ for 15 minutes. Finally, 100 µL of stop solution was added to each well. After mixing thoroughly, the absorbance at 450 nm (OD450) was immediately measured using a microplate reader.

**Flow Cytometry**

For intracellular staining, cells were fixed with a fixation and permeabilization solution (BD Biosciences, 554722). Cells were then incubated with APC anti-mouse CD206 antibody (Biolegend, 141707, 1:100) in the dark at 4℃ for 1 hour. Subsequently, the cells were washed with PBS and resuspended. The stained cells were analyzed using a flow cytometer (BD FACSCelesta, USA). Data were analyzed using FlowJo software (FlowJo LLC, Ashland, OR, USA).

**Immunofluorescence Staining**

The RAW264.7 cells were cultured for 24 hours with or without LPS (200 ng/mL) stimulation and were subsequently stimulated with different concentrations of GLP-1. The ankle joints were collected, and sectioned into slices 10 μm thick. The cells or slices were fixed with 4% paraformaldehyde and permeabilized with 0.5% Triton X-100. The cells were then blocked with a blocking solution (PBS solution with 5% BSA) and incubated overnight at 4℃ with primary antibodies: Anti-CD68 antibody (Abcam, ab53444), CD86 Rabbit Polyclonal Antibody (Proteintech, 13395-1-AP), CD206 Rabbit Polyclonal Antibody (Proteintech, 18704-1-AP), Hexokinase 2 Rabbit Polyclonal Antibody (Proteintech, 22029-1-AP), and VDAC1/Porin Mouse Monoclonal Antibody (Proteintech, 66345-1-Ig-1001). Following DAPI staining, the cells were imaged using an inverted immunofluorescence microscope (Eclipse Ti2, Nikon, Japan).

**Confocal Laser Scanning Microscopy Imaging**

The RAW264.7 cells were cultured for 24 hours with or without LPS (200 ng/mL) stimulation and were subsequently stimulated with GLP-1. After treatment, the cells were fixed with 4% paraformaldehyde and incubated overnight at 4℃ with primary antibodies: Anti-CD68 antibody (Abcam, ab53444), CD86 Rabbit Polyclonal Antibody (Proteintech, 13395-1-AP), and CD206 Rabbit Polyclonal Antibody (Proteintech, 18704-1-AP). After DAPI staining, the stained cells were imaged using the laser scanning confocal microscope (LSM 800, Zeiss, Germany).

**AIA Model**

To construct a rheumatoid arthritis model, 0.1 mL of Freund's complete adjuvant containing 10 mg/mL heat-inactivated mycobacteria (Chondrex, #7027, Washington DC, USA) was subcutaneously injected into the footpad and tail of male BALB/c mice (18-22 g, 6 weeks old). The progression of arthritis was monitored, and the successful establishment of the rheumatoid arthritis model was confirmed on day 14 after injection.

**Assessment of Therapeutic Efficacy**

35 mice were randomly divided into seven groups: (1) the control group; (2) the AIA model group; (3) the sham operation group (simple blue light irradiation); (4) the DEX group (dexamethasone, Veterinary Drug No. 120152530); (5) the 1C hydrogel experimental group (HEK-293 cells number: 9.3×10^5^); (6) the 1/2C hydrogel experimental group (HEK-293 cells number: 4.6×10^5^); and (7) the 1/4C hydrogel experimental group (HEK-293 cells number: 2.3×10^5^). The DEX group was treated with intravenous tail injection of 0.05 mL/g dexamethasone injection solution, administered every three days. The hydrogel experimental groups had the hydrogel placed subcutaneously in the mouse joints, with a dosage of 100 uL, and were irradiated with blue light, twice a day for 15 minutes per session. The entire treatment period lasted for nine days. During the treatment period, the paw thickness of the ankle joint was assessed every two days. Each paw of the mice was scored on a scale of 0 to 4: 0 indicates normal; 1 indicates slight erythema and/or swelling; 2 indicates moderate redness and swelling; 3 indicates severe swelling; and 4 indicates rigidity and inability to bear weight. The scores of the four paws were then summed up. After the completion of the treatment, the mice were euthanized under anesthesia. The thymus and spleen were immediately removed and weighed. The thymus index and spleen index were calculated by dividing the wet weight of the thymus and spleen by the body weight of the mice, respectively.

**Histopathological Examination**

After the treatment, all mice were euthanized. The ankle joints were excised, fixed with 4% paraformaldehyde, and decalcified. The joints were then embedded in paraffin, sectioned at a thickness of 3 μm, and stained with hematoxylin-eosin (HE) and Safranin-O Fast-Green. The staining was observed under an optical microscope.

**Immunohistochemical Analysis and TRAP Assay**

The ankle joints after treatment were fixed with 4% paraformaldehyde and decalcified. The decalcified joints were then embedded in paraffin and sectioned. The sections were stained with an IL-1 beta polyclonal antibody (Proteintech, 26048-1-AP, 1:100). The TRAP staining kit (Bestbio, BB-44212-30ml) was used to stain the sections according to the instructions.

**Micro-CT Imaging**

All mice were euthanized by dislocation after the completion of treatment. The ankle joints of the mice were removed and fixed in a 4% paraformaldehyde solution. The ankle joints were then scanned using an ex vivo computed tomography scanner (InSyTeFLECT/CT, TRIFOIL IMAGING INC., Trifoil Imaging). The scanning parameters were set at a voltage of 42 kV and a current of 1000 uA, with a resolution of 2 mm. Three-dimensional images of the ankle joints and trabeculae were created by reconstructing the data sets.

**Safety Assessment**

After the completion of treatment, all mice were euthanized, and the key organs including the heart, liver, spleen, lungs, and kidneys were immediately removed for histological analysis.

**Detection of Lactate and ATP Concentrations**

After blue light irradiation, the supernatant of HEK293 cells was immediately collected and added to RAW264.7 cells that had been either unstimulated or stimulated with LPS, and then incubated for 8 hours. The supernatant was then collected for lactate and ATP concentration assays. Lactate Assay Kit (Boxbio, AKAC001C) and ATP Assay Kit (Beyotime, S0026) were used to detect the supernatant according to the instructions.

**Detection of Mitochondrial Membrane Potential**

After blue light irradiation, the supernatant of HEK293 cells was immediately collected and added to RAW264.7 cells that had been either unstimulated or stimulated with LPS, followed by incubation for 8 hours. After removing the culture medium, 1 ml of JC-1 staining working solution (Beyotime, C2003S) was added and mixed thoroughly. The cells were then incubated at 37°C in a cell culture incubator for 20 minutes. After incubation, the supernatant was removed, and the cells were washed twice with JC-1 staining buffer, followed by the addition of cell culture medium. The cells were then observed under an inverted fluorescence microscope (Eclipse Ti2, Nikon, Japan).

**Detection of cell apoptosis**

After blue light irradiation, the supernatant of HEK293 cells was immediately collected and added to RAW264.7 cells that had been either unstimulated or stimulated with LPS, followed by incubation for 8 hours. Apoptosis of RAW264.7 cells was detected using the OneStep TUNEL Apoptosis Assay Kit (Meilunbio, MA0223). The cells were then observed under an inverted fluorescence microscope (Eclipse Ti2, Nikon)

**RT-qPCR**

RNA was extracted from cells using Trizol (Takara Bio). Reverse transcription of RNA was performed using the PrimeScript™ RT reagent Kit with gDNA Eraser (TaKaRa, RR047A). Real-time PCR was conducted using TB Green Premix Ex Taq II (Tli RNaseH Plus) on the CFX96 Real-Time PCR Detection System (Bio-Rad). PrimeTime qPCR primer sets were as follows: *HK2*, ATTGTGGCTGTGGTGAA (Forward) and AATGTGACGCATCTCCTC (Reverse); *VDAC1*, CCCACATACGCCGATCTTGG (Forward) and GTGGTTTCCGTGTTGGCAGA (Reverse); *Gapdh*, CCTCGTCCCGTAGACAAAATG (Forward) and TGAGGTCAATGAAGGGGTCGT (Reverse).

**Western blotting**

Cells were lysed using RIPA lysis buffer (Beyotime), and the protein extracts were obtained from the supernatant by high-speed centrifugation. The extracts were subjected to SDS-PAGE and then transferred to a membrane, which was blocked with 5% BSA. The membrane was subsequently incubated overnight with Hexokinase 2 rabbit polyclonal antibody (22029-1-AP, Proteintech), VDAC1/Porin mouse monoclonal antibody (66345-1-Ig-1001, Proteintech), and β-actin antibody (GB15003, Servicebio). Following this, the primary antibodies were incubated with species-appropriate horseradish peroxidase-conjugated secondary antibodies. The membrane was then washed three times with TBST buffer and subjected to immunoblotting using an enhanced chemiluminescent substrate (P1034-200ml, Tianya Bio).

**Ca^2+^ influx experiment**

After treating RAW264.7 cells or RAW264.7 cells pretreated with LPS with GLP-1, 1X Rhod-2AM (IR1880, Solarbio) working solution was added to the cells, and the cells were incubated in a cell culture incubator at 37℃ for 30 minutes. Then, the working solution was washed off with HHBS buffer, and the cells were observed under an inverted fluorescence microscope (Eclipse Ti2, Nikon, Japan).

**Cell Viability Assay**

The hydrogels containing cells were implanted subcutaneously into the thighs of mice and retrieved after 1 day, 5 days, and 9 days, respectively. The hydrogels were washed with PBS to remove impurities from the surface, and then incubated with an appropriate volume of Calcein AM/PI working solution (C2015M, Beyotime) for 30 minutes. The staining effect was observed under an inverted fluorescence microscope (Eclipse Ti2, Nikon, Japan).

**Bioinformatics Analysis**

The raw data was handled by Skewer v0.2.2 and data quality was checked by FastQC v0.11.2. The read length was 2×150 bp. Clean reads were aligned to the mouse genome (mm10) from ensmble using STAR, with one mismatch allowed. StringTie (v1.3.1c) was used to generate gene expression data and differential gene expression was analysed by DESeq2 (v1.16.1). The thresholds for determining DEGs are P < 0.05 and absolute fold change ≥ 2. Then DEGs were chosen for function and signaling pathway enrichment analysis using TopGO and KEGG database. The significantly enriched pathways were determined when P < 0.05.

**Statistical Analysis**

GraphPad Prism 8.0 software and Excel were used for data analysis. Data are presented as the mean ± SD. Prior to significance testing, the normality of data distribution was assessed using the Shapiro-Wilk test, and homogeneity of variances was evaluated using the Brown-Forsythe test (for ANOVA) or the F-test (for Student's t-test). For comparisons between two independent groups, an unpaired two-tailed Student's t-test was applied. For comparisons among three or more groups with a single independent variable, one-way analysis of variance (ANOVA) was conducted, followed by Tukey's post hoc test. For experiments involving two independent variables (e.g., time and treatment), two-way ANOVA was utilized. A p-value of less than 0.05 was considered statistically significant (**P* < 0.05, ***P* < 0.01, ****P* < 0.001, and *****P* < 0.0001).


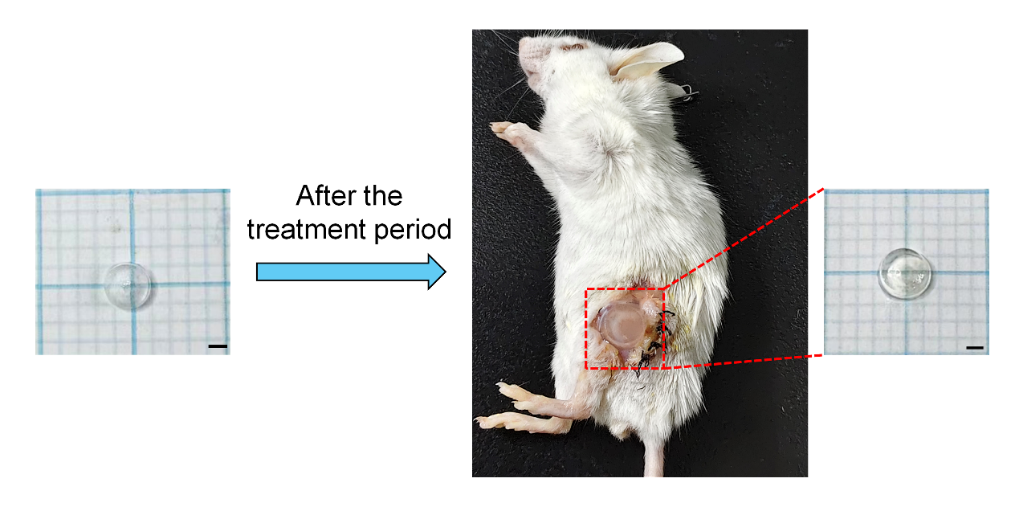


**Figure S1.** The hydrogel morphology variations before and after subcutaneous treatment. Scale bar is 1 mm.


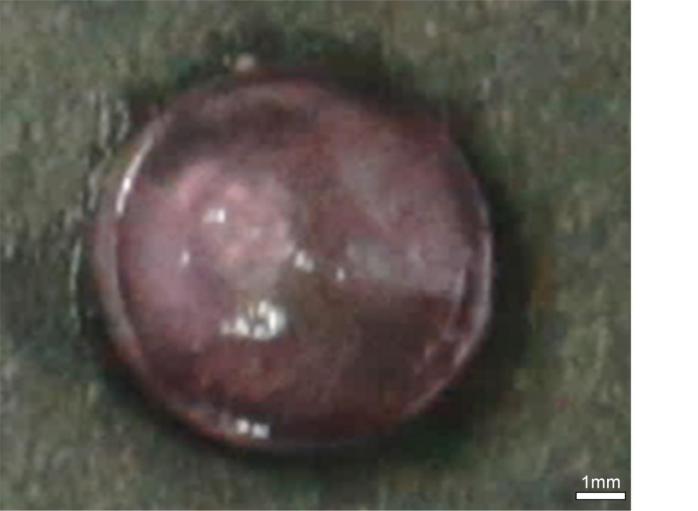


**Figure S2.** A photograph of the hydrogel formulations encapsulating co-transfected HEK-293 cells. Scale bar = 1 mm.


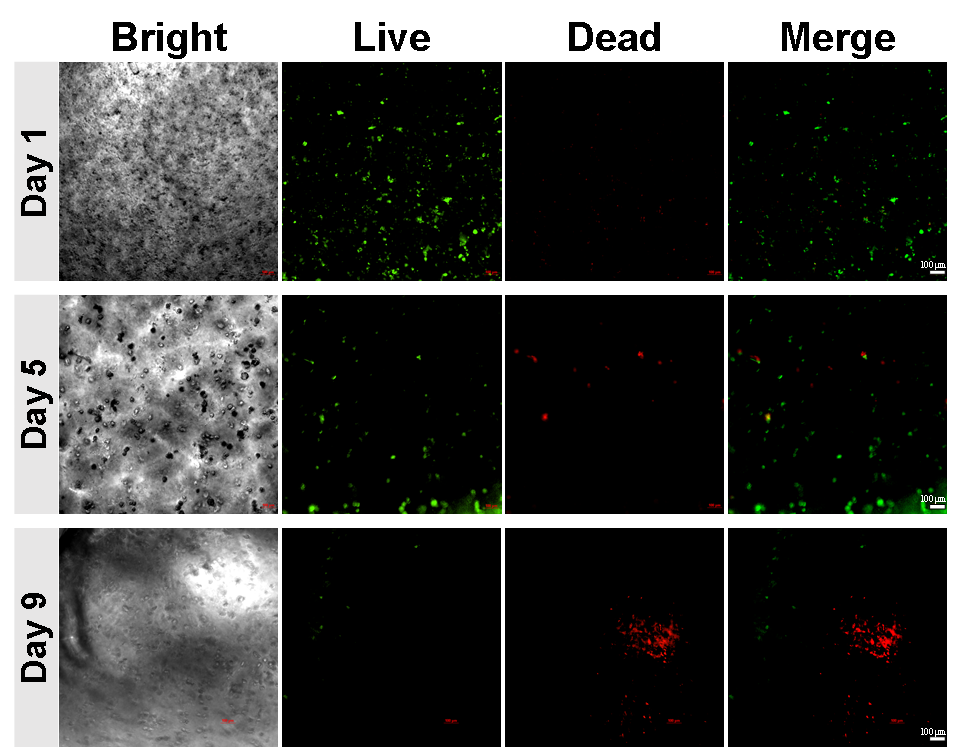


**Figure S3.** Immunofluorescence images showed that the cell viability in the hydrogel on day 1, day 5, and day 9. Green represents live cells, red represents dead cells. Scale bar is 100 μm.


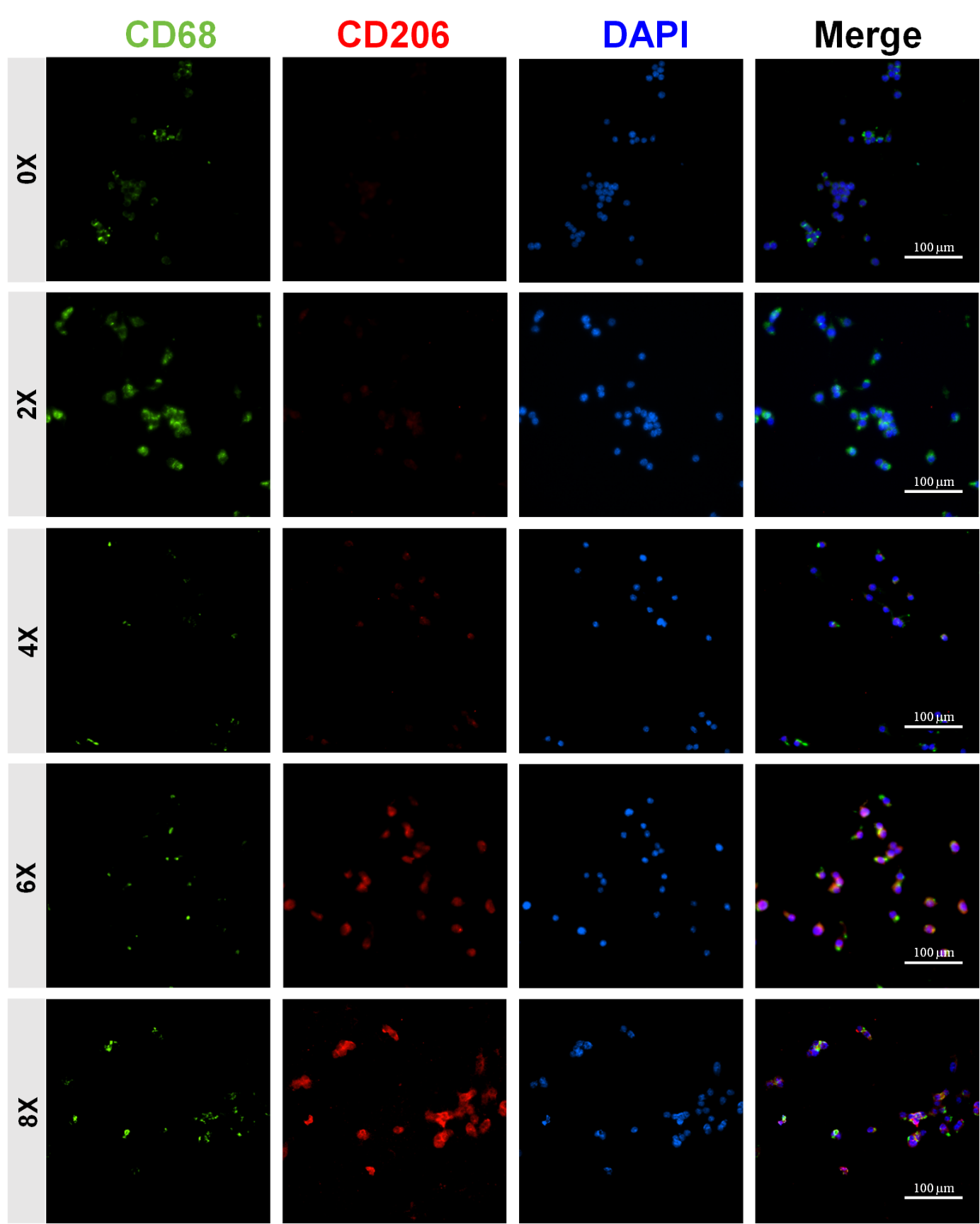


**Figure S4.** Immunofluorescence images showed that with the increasing concentration of GLP-1 stimulation, the expression level of CD206 (red) is enhanced in RAW264.7 cells. CD68 (the pan macrophage marker), CD206 (M2 marker) and DAPI (cell nucleus). Scale bars are 100 μm.


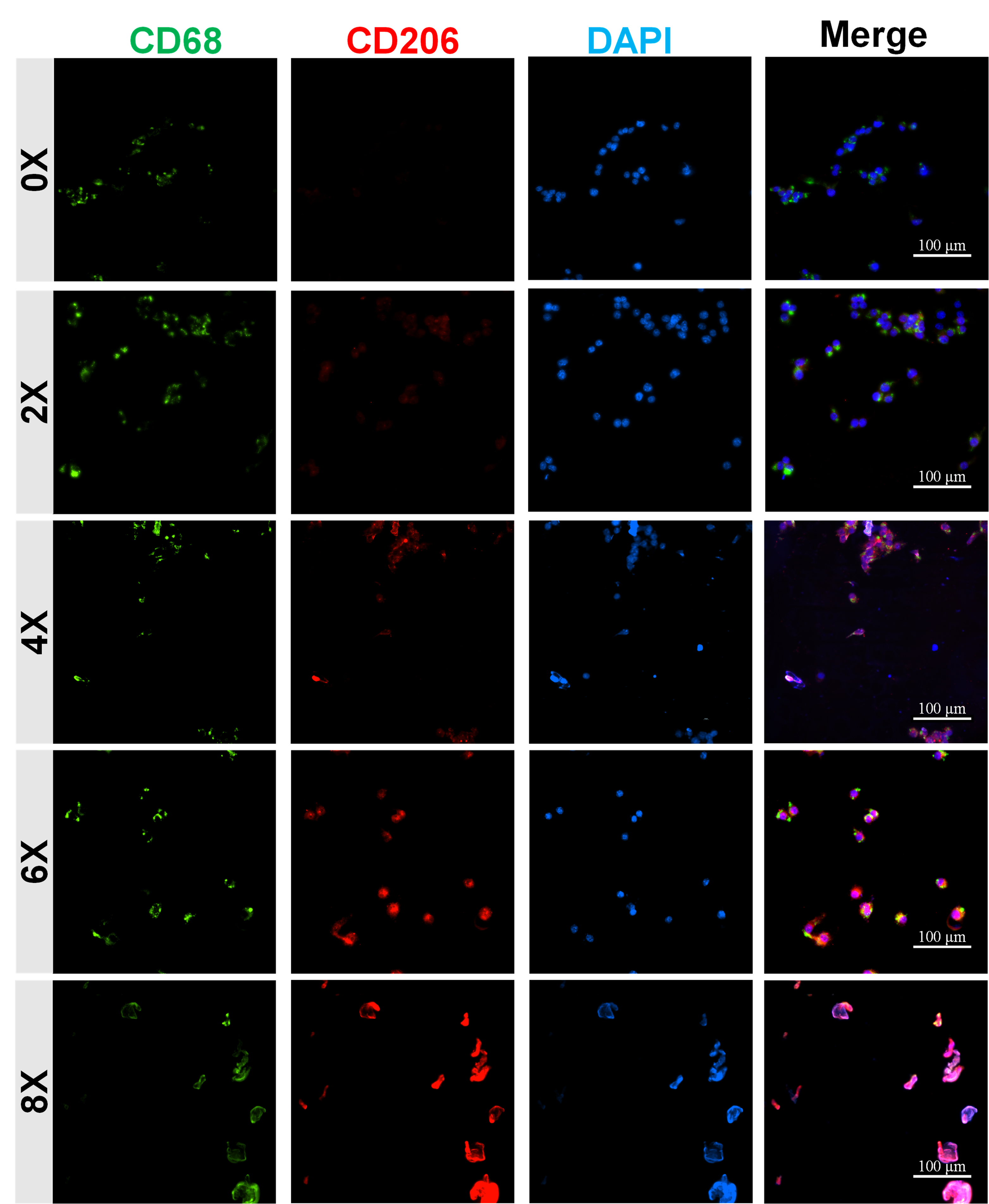


**Figure S5.** Immunofluorescence images showed that with the increasing concentration of GLP-1 stimulation, the expression level of CD206 (red) is enhanced in RAW264.7 cells pretreated with LPS. CD68 (the pan macrophage marker), CD206 (M2 marker) and DAPI (cell nucleus). Scale bars are 100 μm.


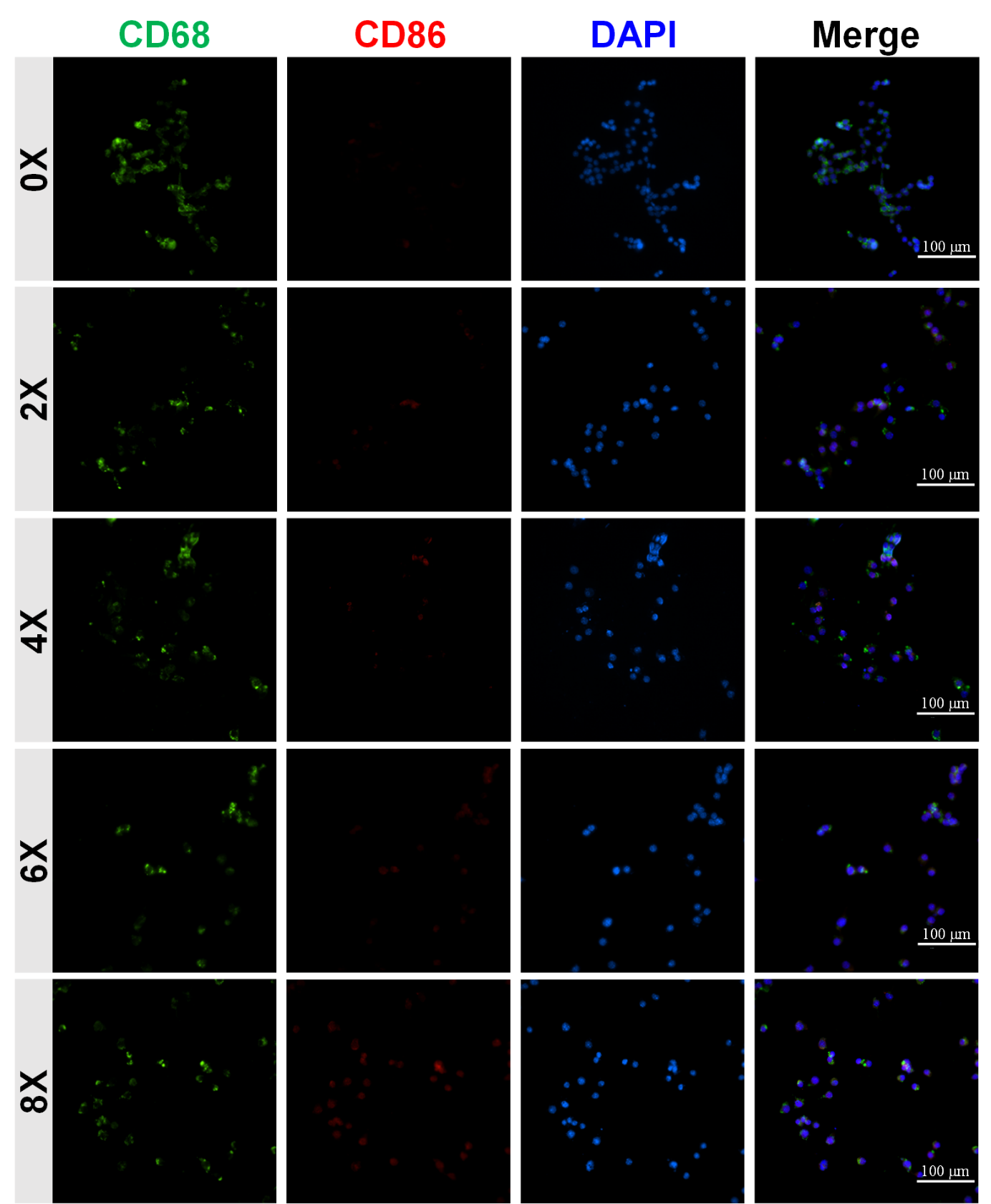


**Figure S6.** Immunofluorescence images showed that with the increasing concentration of GLP-1 stimulation, the expression level of CD86 (red) is detected in RAW264.7 cells. CD68 (the pan macrophage marker), CD86 (M1 marker) and DAPI (cell nucleus). Scale bars are 100 μm.


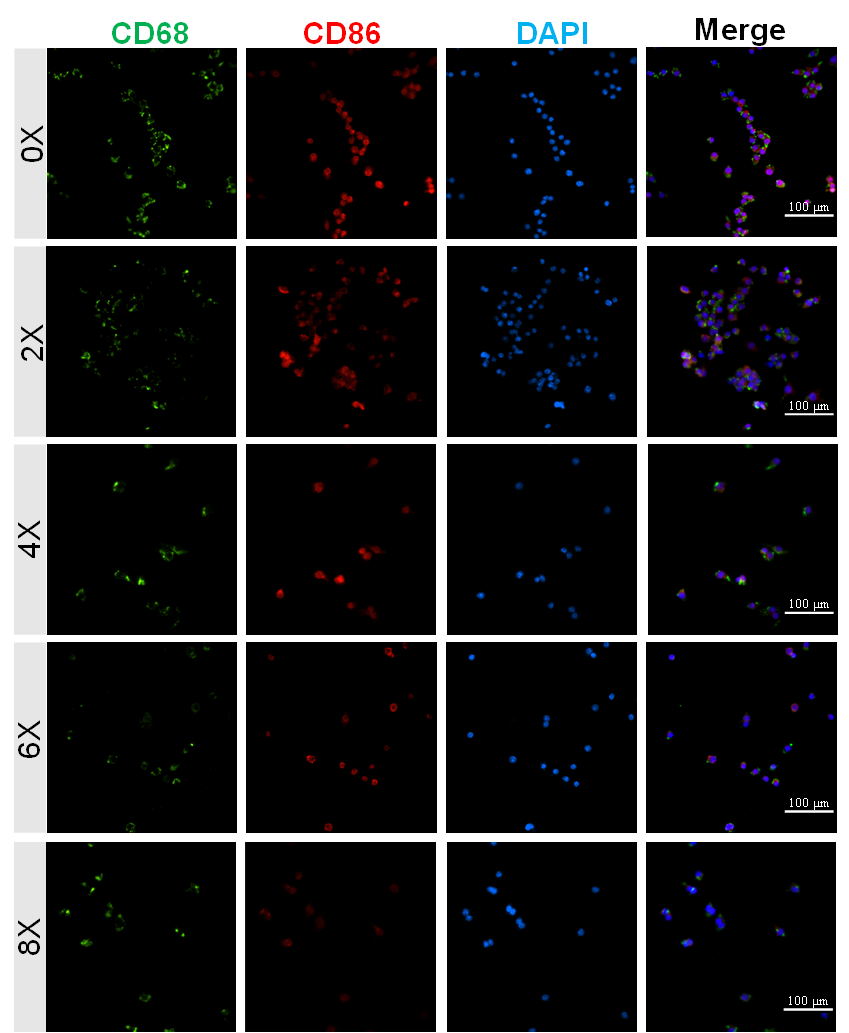


**Figure S7.** Immunofluorescence images showed that with the increasing concentration of GLP-1 stimulation, the expression level of CD86 (red) is detected in RAW264.7 cells pretreated with LPS. CD68 (the pan macrophage marker), CD86 (M1 marker) and DAPI (cell nucleus). Scale bars are 100 μm.


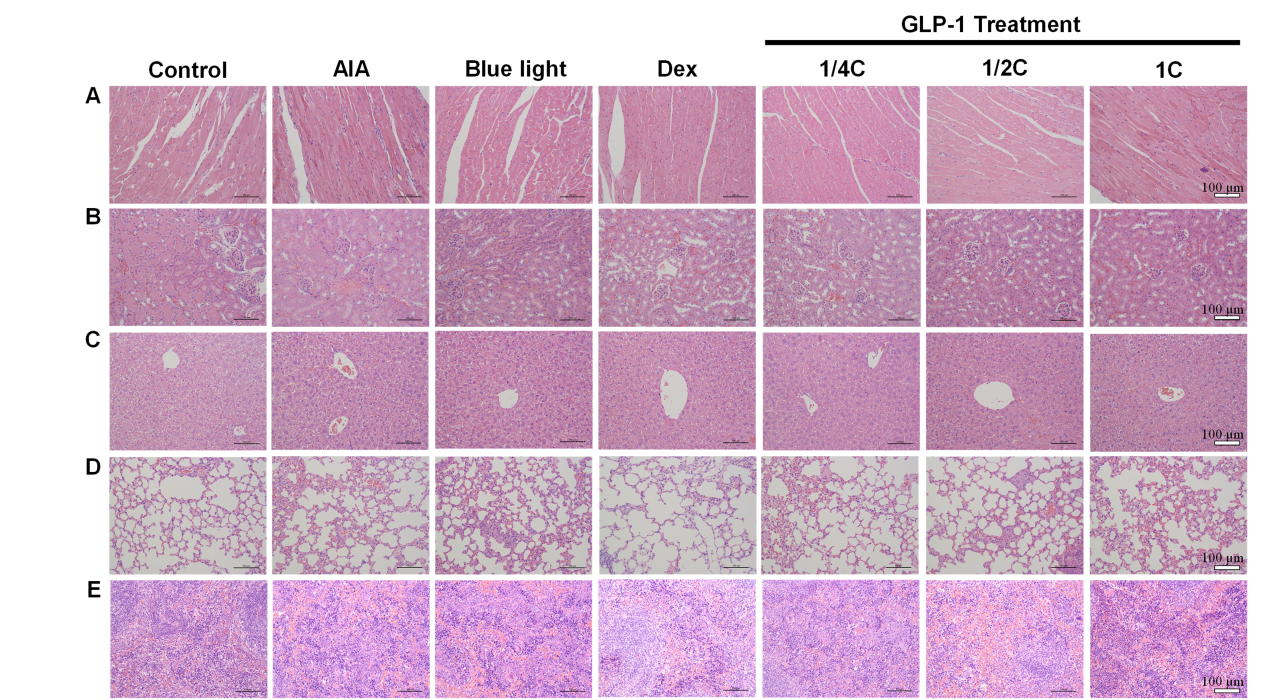


**Figure S8.** Histopathology evaluation of heart (A), kidney (B), liver (C), lung (D), and spleen (E) in mice from different treatment groups were identified using H&E. Scale bars are 100 μm.

**Figure S9**. Body weights of mice in each treatment group before and after treatment. Data represent mean ± SD (n = 5). Data were calculated by two-way ANOVA. ns: not significant.


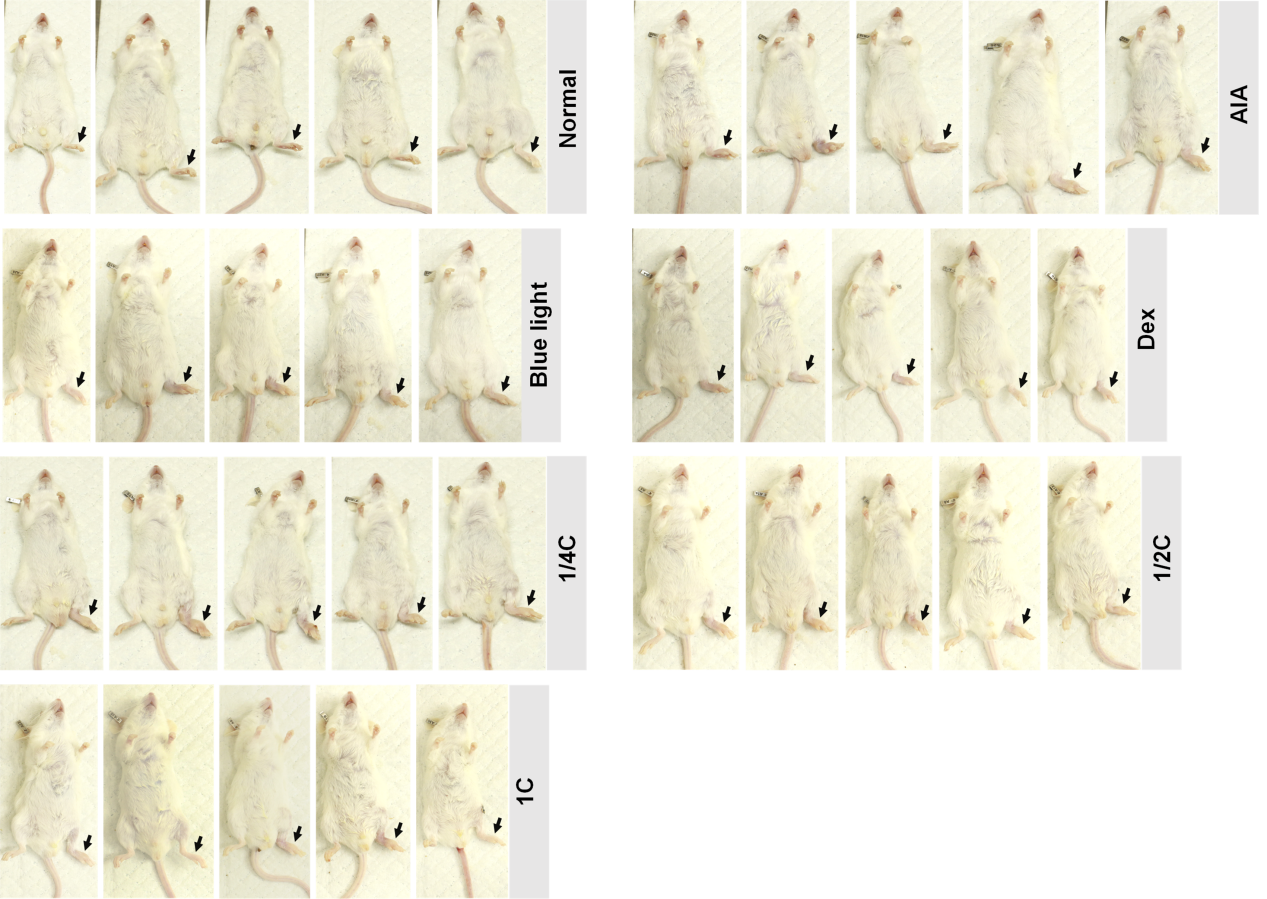


**Figure S10.** Images of the AIA mice in different treatment groups at the end of treatment.


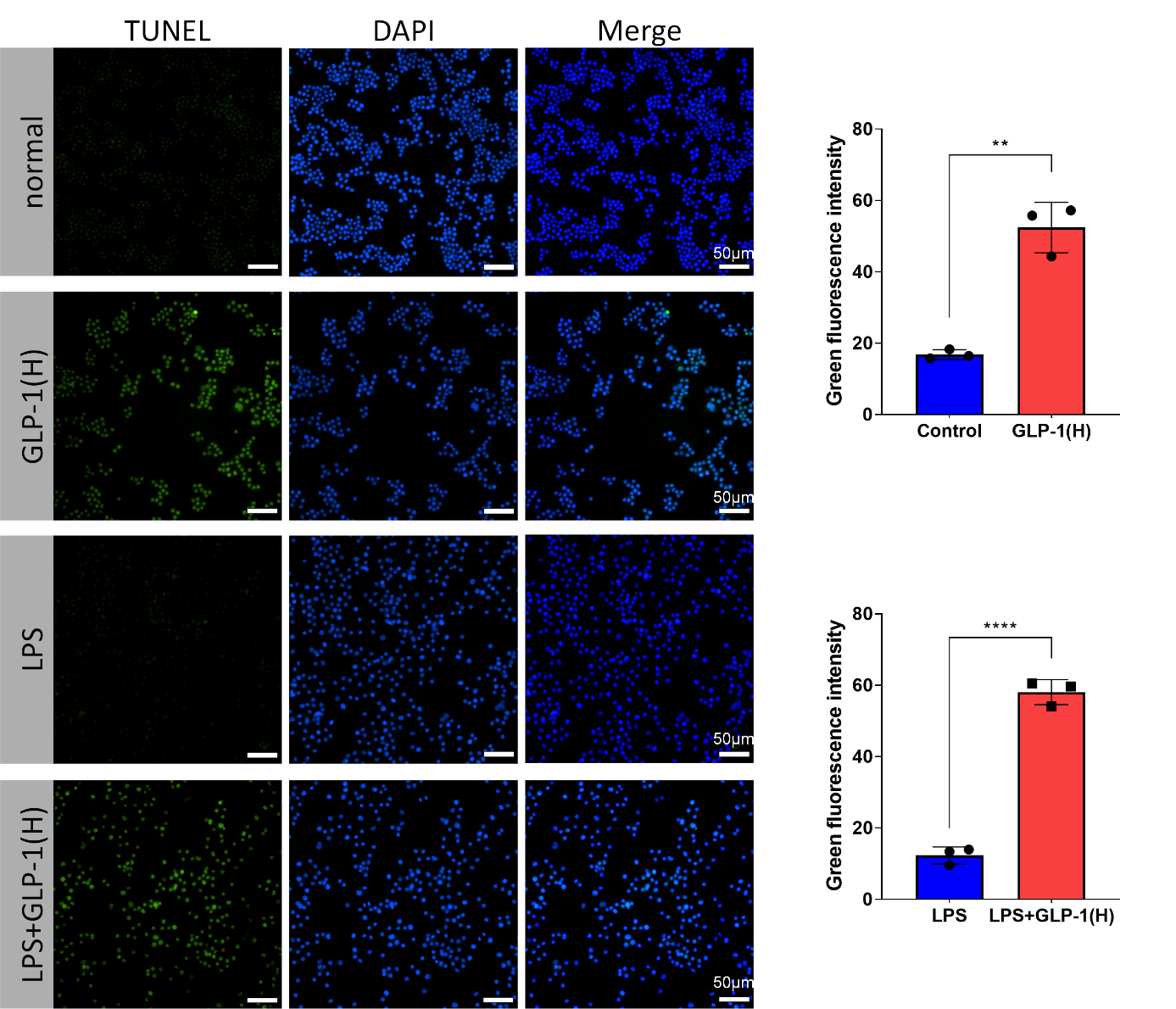


**Figure S11.** Effect of single high-concentration GLP-1 treatment on RAW264.7 cells. Representative TUNEL staining images (Green: TUNEL-positive apoptotic cells; Blue: DAPI-stained nuclei), and the quantification of fluorescence intensity. Scale bars are 50 μm. Data were expressed as mean ± SD, n = 3. Data were calculated by the unpaired two-tailed Student’s t-test. ***P* <0.01, *****P* < 0.0001.


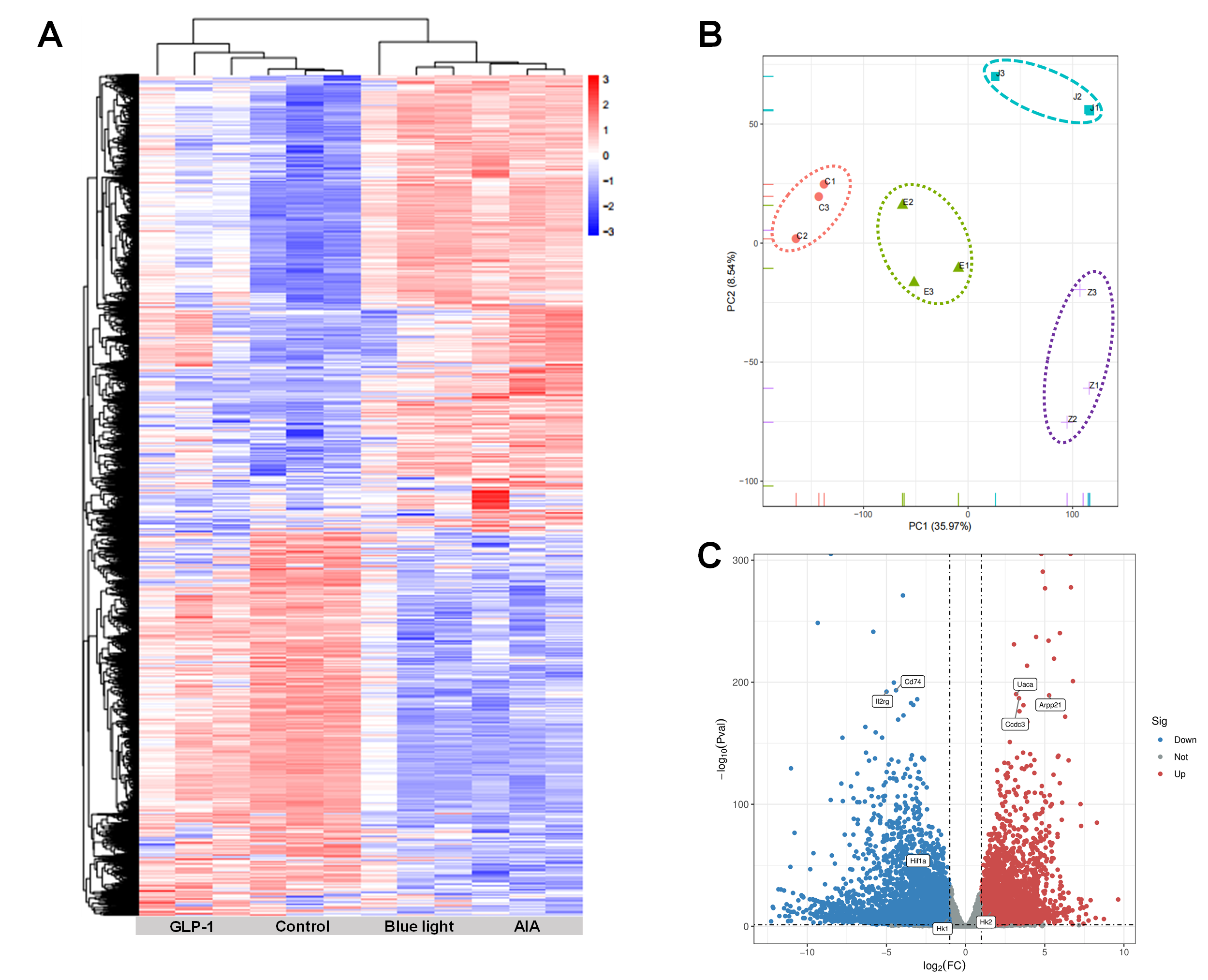


**Figure S12.** (A) Heatmap illustrates the differences in mRNA expression levels among different treatment groups. (B) Principal component analysis of the different treatment groups. (C) Volcano plot of all genes identified in this study. Red dots and blue dots represent significantly upregulated and downregulated genes, respectively.


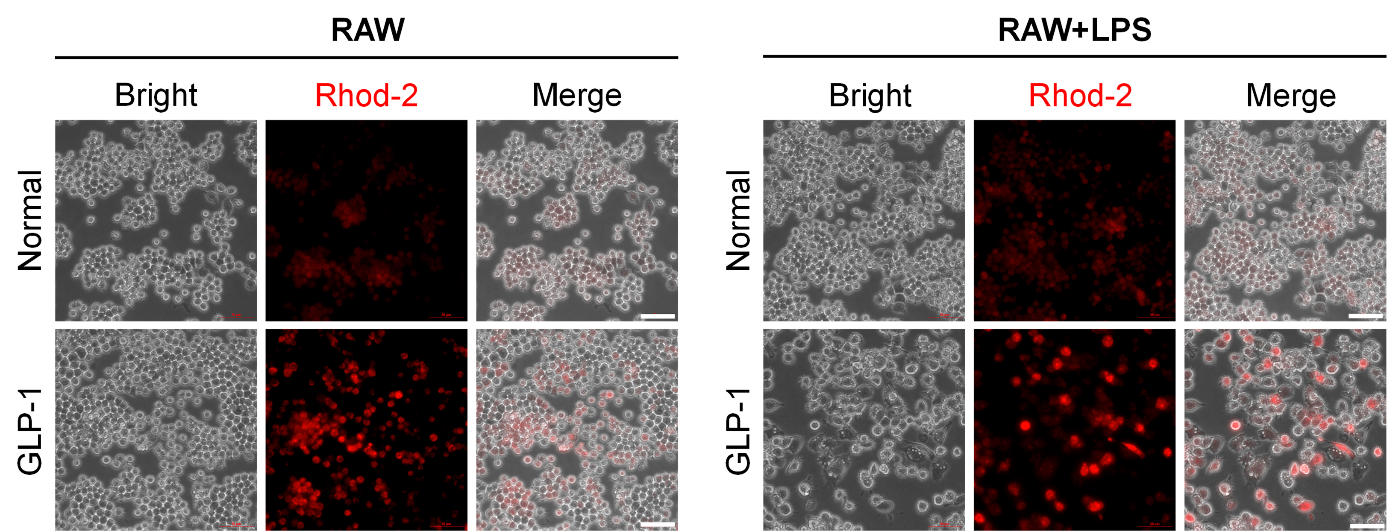


**Figure S13.** Fluorescent images of RAW cells with (left panel) and without LPS treatment (right panel) to indicate the Ca^2+^ influx inducing by GLP-1 treatment. Scale bars are 50 μm.
